# Two distinct trophectoderm lineage stem cells from human pluripotent stem cells

**DOI:** 10.1016/j.jbc.2021.100386

**Published:** 2021-02-05

**Authors:** Adam Mischler, Victoria Karakis, Jessica Mahinthakumar, Celeste K. Carberry, Adriana San Miguel, Julia E. Rager, Rebecca C. Fry, Balaji M. Rao

**Affiliations:** 1 Department of Chemical and Biomolecular Engineering, North Carolina State University, Raleigh, North Carolina, USA; 2 Department of Environmental Sciences and Engineering, Institute for Environmental Health Solutions, University of North Carolina - Chapel Hill, Chapel Hill, North Carolina, USA; 3 Golden LEAF Biomanufacturing Training and Education Center (BTEC), North Carolina State University, Raleigh, North Carolina, USA

**Keywords:** trophoblast, embryonic stem cell, induced pluripotent stem cell, placenta, differentiation, trophoblast stem cells, extravillous trophoblast, syncytiotrophoblast, BSA, bovine serum albumin, CTB, cytotrophoblast, dhS1P, dihydrospingosine-1-phosphate, DMEM, Dulbecco's modified Eagle's medium, EGF, epidermal growth factor, EVT, extravillous trophoblast, FGF, fibroblast growth factor, GO, gene ontology, hESC, human embryonic stem cell, hiPSC, human induced pluripotent stem cell, hPSC, human pluripotent stem cell, KSR, knockout serum replacement, PCA, Principal component analysis, ROCK, RhoA associated kinase, S1P, sphingosine-1 phosphate, S1PR, S1P receptor, STB, syncytiotrophoblast, TGFβ, transforming growth factor-beta, TS, trophoblast stem, TSCM, trophoblast stem cell medium

## Abstract

The trophectoderm layer of the blastocyst-stage embryo is the precursor for all trophoblast cells in the placenta. Human trophoblast stem (TS) cells have emerged as an attractive tool for studies on early trophoblast development. However, the use of TS cell models is constrained by the limited genetic diversity of existing TS cell lines and restrictions on using human fetal tissue or embryos needed to generate additional lines. Here we report the derivation of two distinct stem cell types of the trophectoderm lineage from human pluripotent stem cells. Analogous to villous cytotrophoblasts *in vivo*, the first is a CDX2^-^ stem cell comparable with placenta-derived TS cells—they both exhibit identical expression of key markers, are maintained in culture and differentiate under similar conditions, and share high transcriptome similarity. The second is a CDX2^+^ stem cell with distinct cell culture requirements, and differences in gene expression and differentiation, relative to CDX2^-^ stem cells. Derivation of TS cells from pluripotent stem cells will significantly enable construction of *in vitro* models for normal and pathological placental development.

Specification of the trophectoderm and the inner cell mass is the first differentiation event during human embryonic development. The trophectoderm mediates blastocyst implantation in the uterus and is the precursor to all trophoblast cells in the placenta. Upon embryo implantation, the trophectoderm forms the cytotrophoblast (CTB), a putative stem cell that can differentiate to form the two major cell types in the placenta, the extravillous trophoblast (EVT) and the syncytiotrophoblast (STB) (1, 2). The EVTs are involved in remodeling of uterine arteries, which is critical to ensure adequate perfusion of the placenta with maternal blood, whereas the multinucleated STB mediates the nutrient and gas exchange at the maternal–fetal interface (3, 4). Abnormalities in trophoblast development are associated with pregnancy-related pathologies such as miscarriage, preeclampsia, and placenta accreta. Yet, despite its relevance to maternal and fetal health, constraints on research with human embryos and early fetal tissue impede mechanistic insight into early trophoblast development.

Trophoblast stem (TS) cells derived from first-trimester human placental samples and blastocyst-stage embryos have emerged as an attractive *in vitro* model system for early human trophoblast (5). However, restricted accessibility of embryos and placental samples from early gestation and low genetic diversity of existing cell lines limit the use of this model. In contrast, human pluripotent stem cells (hPSCs) are a more accessible source for generating *in vitro* models of human trophoblast. Of more importance, unlike early gestation primary samples where the projected pregnancy outcome is uncertain, human induced pluripotent stem cells (hiPSCs) can potentially provide models of validated normal and pathological trophoblast development (6). However, whether *bona fide* trophoblast can be obtained from hPSCs has been a subject of intense debate (7). A rigorous head-to-head comparison between trophoblast derived from hPSCs and their *in vivo* counterparts has proven difficult owing to multiple reasons. Previous studies have used varying experimental protocols (8); both primary placental samples and cultures of terminally differentiated trophoblast obtained from hPSCs exhibit heterogeneity and contain many cell types, and until recently self-renewing TS-like cells had not been derived from hPSCs (9, 10, 11, 12).

In this study, we report the derivation and maintenance of two distinct trophectoderm lineage stem cell types from hPSCs, specifically human embryonic stem cells (hESCs) and hiPSCs, in chemically defined culture conditions. The first is a CDX2^-^ stem cell that is comparable with TS cells derived from early-gestation placental samples and similar to the villous CTB. The second is a CDX2^+^ cell type with distinct cell culture requirements, and differences in gene expression and differentiation, relative to CDX2^-^ stem cells. Critically, the isolation of self-renewing stem cell populations allowed a direct comparison of placenta-derived TS cells with TS cells from hPSCs; genome-wide transcriptomic analysis and functional differentiation assays demonstrate very high similarity between placenta- and hPSC-derived CDX2^-^ TS cells. The routine derivation of TS cells from hPSCs will provide powerful tools for mechanistic studies on normal and pathological early trophoblast development.

## Results

### A chemically defined medium containing sphingosine-1 phosphate enables differentiation of hESCs to CTB

**Figure 1:**
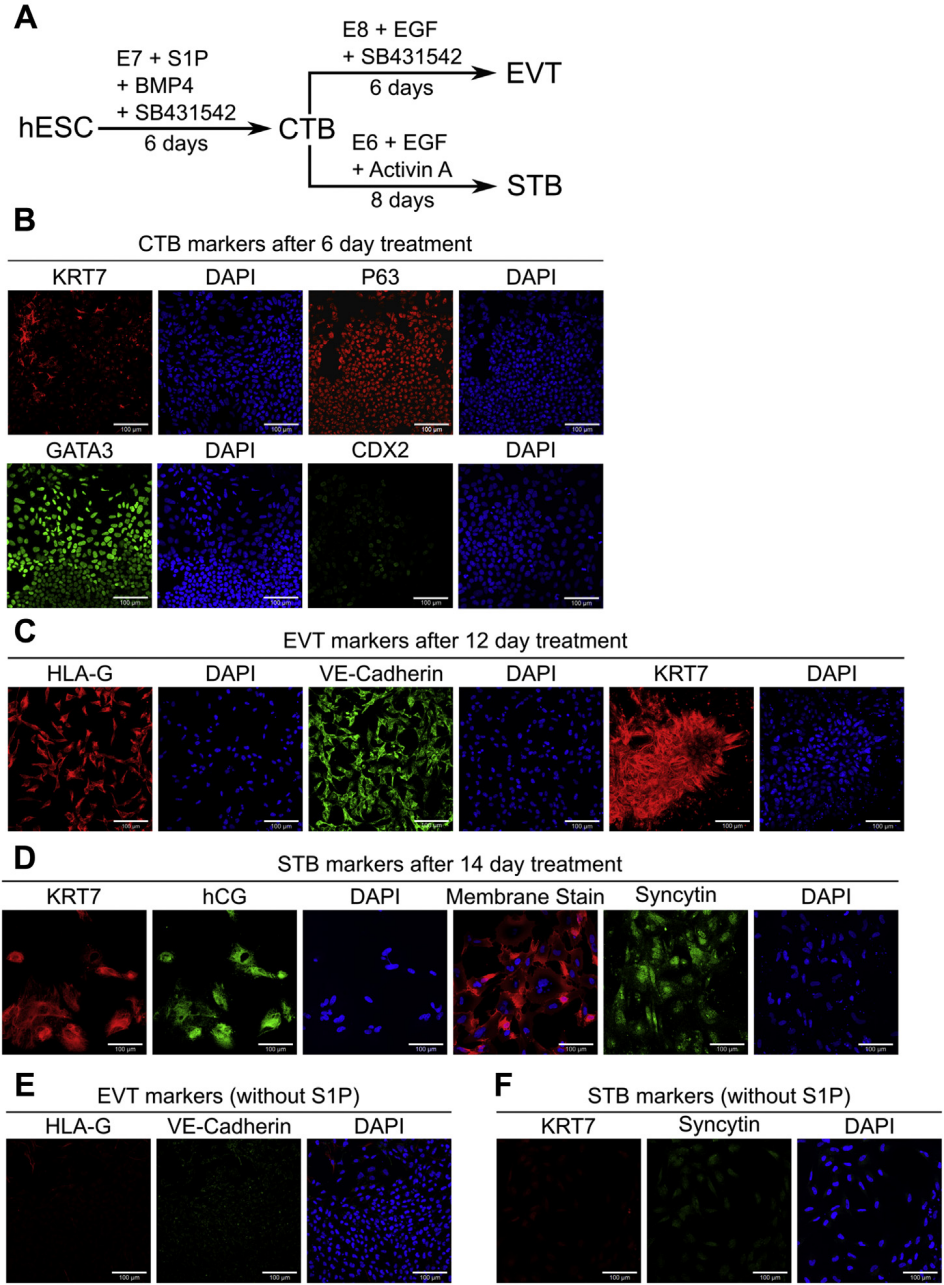
**A chemically defined medium containing S1P enables differentiation of hESCs to CTB-like cells and terminally differentiated trophoblasts.***A*, schematic of protocol for hESC differentiation to trophoblast. *B*, confocal images of CTB from 6-day initial treatment of H9 hESCs, staining for KRT7, P63, GATA3, and CDX2. Nuclei were stained with DAPI. *C*, confocal images of EVTs from 12-day treatment of H9 hESCs, staining for KRT7, HLA-G, and VE-Cadherin. Nuclei were stained with DAPI. *D*, confocal images of STB from 14-day treatment of H9 hESCs, staining for KRT7 and hCG, and syncytin. Nuclei were stained with DAPI. Membrane was stained with CellMask deep *red* plasma membrane stain. *E*, confocal images of cells from 12-day EVT treatment of H9 hESCs upon removal of S1P, staining for HLA-G and VE-Cadherin. Nuclei were stained with DAPI. *F*, confocal images of cells from 14-day STB treatment of H9 hESCs upon removal of S1P, staining for KRT7 and syncytin. Nuclei were stained with DAPI. The *scale bars* represent 100 μm for all images. CTB, cytotrophoblast; DAPI, 4′,6-diamidino-2-phenylindole; EGF, epidermal growth factor; EVT, extravillous trophoblast; hESC, human embryonic stem cell; S1P, sphingosine-1 phosphate; STB, syncytiotrophoblast.

Media formulations in previous studies on trophoblast differentiation of hESCs included components such as knockout serum replacement (KSR) or bovine serum albumin (BSA) that act as carriers for lipids. Albumin-associated lipids have been implicated in activation of G-protein–coupled receptor–mediated signaling (13, 14). For instance, the phospholipid sphingosine-1 phosphate (S1P) present in KSR can activate YAP signaling. YAP plays a critical role in specification of the trophectoderm in mouse (15, 16, 17), as well as human trophoblast development (18, 19). We investigated the use of S1P in the context of trophoblast differentiation of hESCs under chemically defined culture conditions, by modifying our previous protocol that utilized KSR (20, 21). H1 and H9 hESCs cultured in E8 medium were differentiated for 6 days in E7 medium (E8 without transforming growth factor-beta1 [TGFβ1]) supplemented with S1P, by treatment with BMP4 and the activin/nodal inhibitor SB431542 (Fig. 1*A*). Under these conditions, we observed upregulation of the trophectoderm marker *CDX2* and the CTB marker *ELF5* (Fig. S1, *A* and *B*). Upregulation of *TBX4* was observed after 6 days. However, overall there were no significant changes in markers associated with neural or mesodermal differentiation after 6 days suggesting that differentiation to these lineages did not occur (Fig. S1, *A* and *B*). Immunofluorescence analysis at day 6 confirmed expression of the pan-trophoblast marker KRT7, and CTB markers P63 and GATA3; expression of CDX2 was not observed (Figs. 1*B* and S1*C*).

The putative CTB cells obtained at day 6 were investigated for their ability to differentiate to EVTs and STB, using protocols similar to those previously employed (20). We observed formation of mesenchymal cells from epithelial cells over a 6-day period when passaged into E8 medium supplemented with epidermal growth factor (EGF) and SB431542. Immunofluorescence analysis showed expression of KRT7 and the EVT markers VE-Cadherin and HLA-G (Figs. 1*C*, S1*D*). Alternatively, passaging CTB-like cells in E6 medium (E8 without TGFβ1 and fibroblast growth factor-2 [FGF2]) supplemented with activin and EGF resulted in the formation of KRT7^+^ multinucleate cells expressing the STB markers hCG and syncytin over an 8-day period (Figs. 1*D*, S1*E*). Removal of S1P from the medium during hESC differentiation to CTB-like cells abolished the formation of EVTs that express HLA-G and VE-Cadherin (Figs. 1*E*, S2*A*) under identical differentiation conditions (Fig. 1*A*). Differentiation to STB also did not occur in the absence of S1P, as evidenced by lack of expression of syncytin and KRT7 (Figs. 1*F*, S2*B*). Also, downregulation of the trophectoderm marker *CDX2* and upregulation of transcripts of neural and mesoderm markers was observed in cells after 6 days of differentiation, upon removal of S1P (Fig. S2*C*). Taken together these results show that CTB-like cells, similar to those in previous studies utilizing more complex culture conditions (20), can be obtained by differentiation of hESCs in a chemically defined medium containing S1P. Furthermore, addition of exogenous S1P is necessary for hESC differentiation to trophoblast in our chemically defined culture medium.

Rho GTPase signaling, downstream of G-protein–coupled receptors activated by S1P, has been implicated in nuclear localization of YAP (22, 23). Both Rho/RhoA associated kinase (ROCK) and nuclear YAP play a critical role in trophectoderm specification in the mouse (24, 25). Therefore, we investigated the role of Rho/ROCK signaling and YAP in trophoblast differentiation of hESCs. The Rho/ROCK inhibitor Y-27632 was included during differentiation of hESCs to CTB-like cells and subsequent differentiation to EVT and STB to investigate the role of Rho/ROCK signaling. Under these conditions, HLA-G expression was observed in cells obtained from H9 hESCs; however, VE-Cadherin expression was weak and observed in only a few cells (Fig. S3*A*). On the other hand, expression of EVT markers was not observed in cells derived from H1 hESCs. In addition, presence of ROCK inhibition abolished STB formation, as shown by the lack of expression of syncytin and KRT7 (Fig. S3*B*).

To investigate the role of YAP signaling in CTB formation from hESCs, we used an hESC cell line (H9) that expresses an inducible shRNA against YAP (H9-YAP-ishRNA) or a scrambled shRNA control (26). YAP knockdown abolished differentiation to EVT and STB, as evidenced by lack of expression of the relevant markers. It is notable that high cell death was observed (Fig. S3, *A* and *B*). Gene expression analysis revealed a significant reduction in *ELF5* upon YAP knockdown, relative to the scrambled shRNA control (Fig. S3*C*). Significant downregulation of the mesodermal genes *TBX4* and *LMO2* was observed, whereas *T* was upregulated, in H9-YAP-ishRNA, relative to the scrambled control. Taken together, these results show that Rho/ROCK signaling and YAP are necessary for differentiation of hESCs to functional CTB that can give rise to both EVTs and STB, in our chemically defined culture medium.

### S1P mediates its effects on trophoblast differentiation of hESCs through its receptors

**Figure 2:**
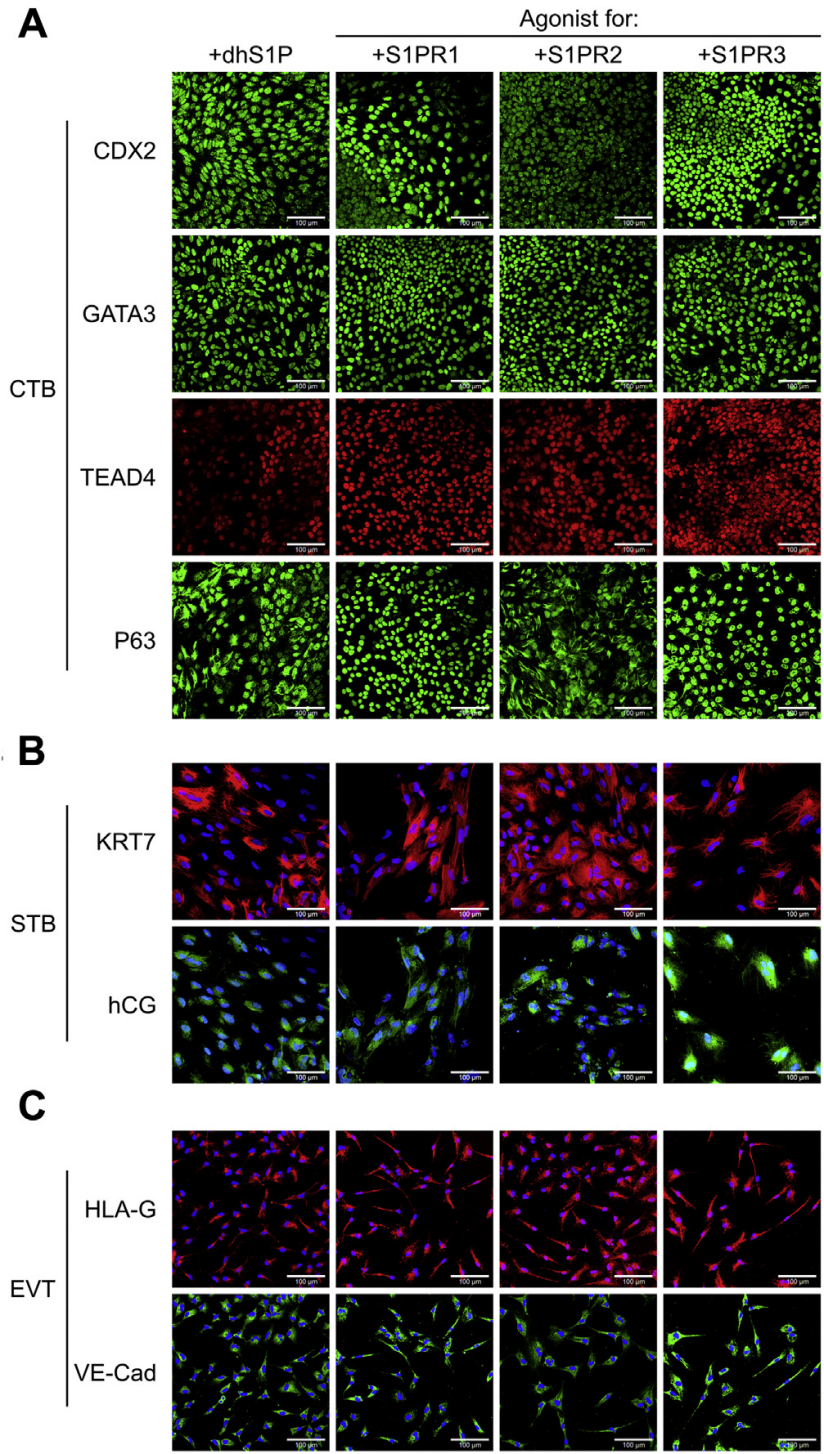
**Sphingosine-1 phosphate mediates its effects on trophoblast differentiation of hESCs through its receptors**. *A*, confocal images of CTB from 6-day treatment of H9 hESCs using D-erythro-dihydrospingosine-1-phosphate (dhS1P), CYM5442 (S1PR1 agonist), CYM5220 (S1PR2 agonist), and CYM5541 (S1PR3 agonist), staining for CDX2, GATA3, P63, and TEAD4. Nuclei were stained with DAPI. *B*, confocal images of STB from 14-day treatment of H9 hESCs using dhS1P, CYM5442, CYM5520, and CYM5541 during initial 6-day treatment, staining for KRT7 and hCG. Nuclei were stained with DAPI. *C*, confocal images of EVTs from 12-day treatment of H9 hESCs using dhS1P, CYM5442, CYM5220, and CYM5541 during initial 6-day treatment, staining for HLA-G and VE-Cadherin. Nuclei were stained with DAPI. The scale bars represent 100 μm for all images. CTB, cytotrophoblast; DAPI, 4′,6-diamidino-2-phenylindole; EVT, extravillous trophoblast; hESC, human embryonic stem cell; STB, syncytiotrophoblast.

S1P acts through both receptor-mediated and receptor-independent pathways (14, 27). To investigate the specific mechanism of S1P action during hESC differentiation to trophoblast, we replaced S1P with D-erythro-dihydrospingosine-1-phosphate (dhS1P) in our protocol. dhS1P acts as an agonist for the S1P receptors (S1PRs) but does not mediate an intracellular effect (28). Replacing S1P with dhS1P yielded similar results—CTB-like cells showed expression of CDX2, GATA3, P63, and TEAD4 (Figs. 2*A* and S4*A*). Upon further differentiation as previously described (Fig. 1*A*), STB expressing KRT7 and hCG, and EVT expressing HLA-G and VE-Cadherin were obtained (Fig. 2, *B* and *C*; Fig. S4, *B* and *C*). These results suggest that S1PR signaling mediates the effect of exogenous S1P during hESC differentiation to trophoblast in our chemically defined medium.

S1P acts extracellularly through S1PR1-5 (14, 27); however, TBs have been shown to only express S1PR1-3 (29). We further used selective chemical agonists for S1PR1-3—CYM5442 hydrochloride, CYM5520, and CYM5541, respectively—to replace S1P in differentiation protocols previously discussed. Expression of CDX2, GATA3, P63, and TEAD4 was observed in CTB-like cells for all three agonists (Figs. 2*A* and S4*A*). Similarly, use of each agonist resulted in expression of the EVT markers HLA-G and VE-Cadherin and formation of multinucleate STB expressing KRT7 and hCG (Fig. 2, *B* and *C*; Fig. S4, *B* and *C*). However, we observed some variability between the agonists (Fig. S5). For instance, use of the S1PR2 agonist resulted in strong cytoplasmic expression of P63 and high heterogeneity in staining at day 6 relative to the other agonists. Formation of large multinucleated STB was more pronounced when the S1PR2 or S1PR3 agonists were used, as compared with the S1PR1 agonist. On the other hand, the S1PR1 and S1PR3 agonists enhanced the formation of mesenchymal EVTs, relative to the S1PR2 agonist. Taken together, our results further confirmed that S1PR signaling mediates effects of exogenous S1P during trophoblast differentiation of hESCs in our culture system. Since our qualitative observations showed that use of the S1PR3 agonist resulted in expression of CTB markers, and both multinucleate STB and mesenchymal EVTs could be obtained when the S1PR3 agonist was used, we chose the S1PR3 agonist for subsequent studies.

### Optimizing timing of hESC differentiation enables derivation of CDX2^+^ TS cells

**Figure 3:**
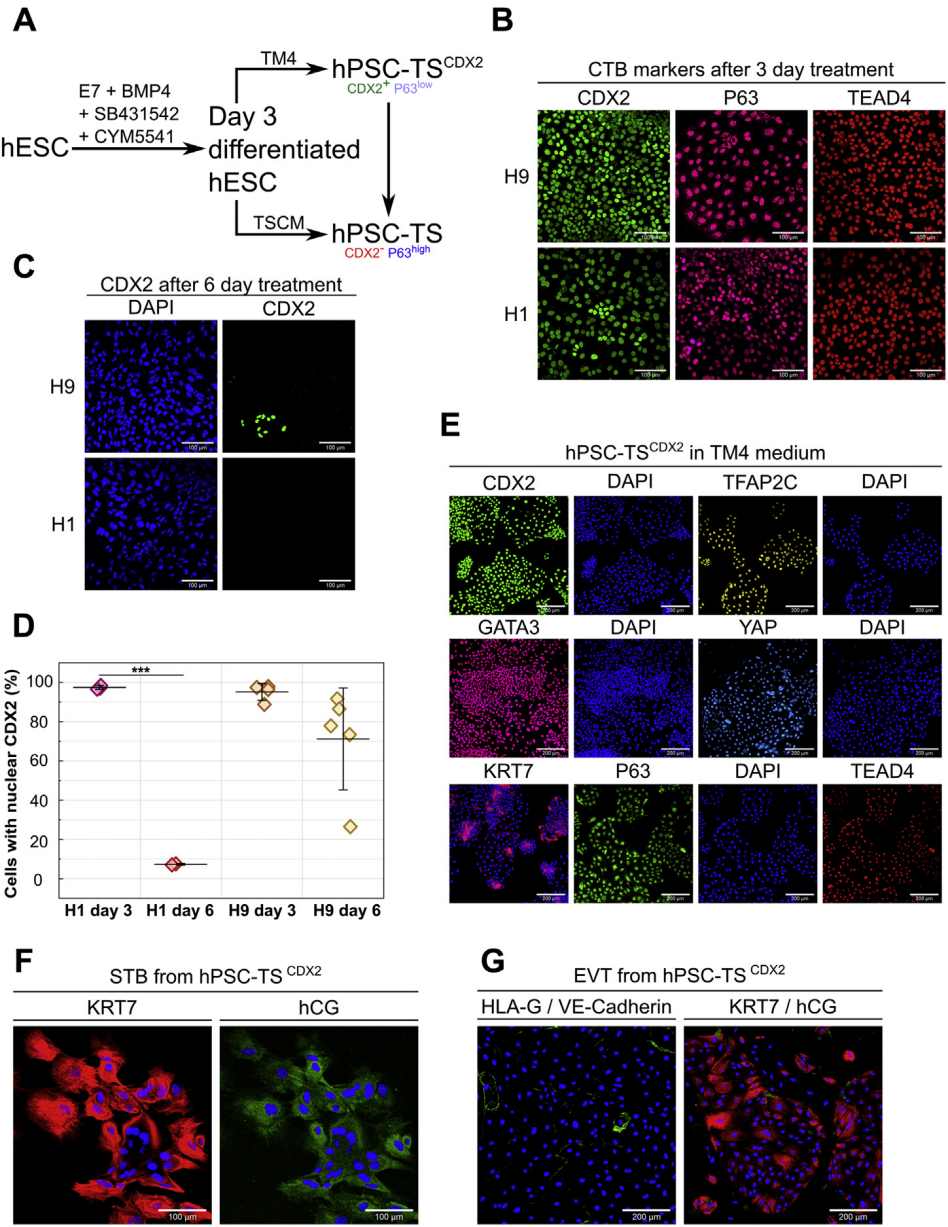
**Optimizing timing of hESC differentiation enables derivation of hPSC-TS**^**CDX2**^**cells**. *A*, schematic of differentiation protocol for establishment of hPSC-TS^CDX2^ and hPSC-TS from hESCs. *B*, confocal images of 3 days treated H9 and H1 hESCs, staining for CDX2, P63, and TEAD4. Nuclei were stained with DAPI. The scale bars represent 100 μm. *C*, confocal images of 6 days treated H9 and H1 hESCs, staining for CDX2. Nuclei were stained with DAPI. The scale bars represent 100 μm. *D*, quantitative analysis of cells expressing nuclear CDX2 after 3- and 6-day differentiation treatment of H1 (day 3, 5455 cells in three images; day 6, 2448 cells in two images) and H9 (day 3, 5552 cells in four images; day 6, n = 6448 cells in five images) hESCs. Data points represent fraction of CDX2^+^cells in individual images from at least two biological replicates. Analysis was performed in MATLAB and at least two biological replicates were used. (Error bars are SD, ∗∗∗*p* < 0.05). *E*, confocal images of H9 hPSC-TS^CDX2^ in TM4, staining for CDX2, TFAP2C, GATA3, YAP, TEAD4, and P63. Nuclei were stained with DAPI. The scale bars represent 200 μm. *F*, confocal images of STB from H9 hPSC-TS^CDX2^ staining for hCG and KRT7. Nuclei were stained with DAPI. The scale bars represent 100 μm. *G*, confocal images of EVTs from H9 hPSC-TS^CDX2^, staining for HLA-G (*red*) and VE-Cadherin (*green*) as well as KRT7 (*red*) and hCG (*green*). Nuclei were stained with DAPI. The scale bars represent 200 μm. DAPI, 4′,6-diamidino-2-phenylindole; hESC, human embryonic stem cell; hPSC, human pluripotent stem cell; STB, syncytiotrophoblast; TS, trophoblast stem.

We investigated whether CTB-like cells obtained by treatment of hESCs with BMP4 and SB431542 in E7 medium supplemented with the S1PR3 agonist CYM5541 for 6 days could be passaged and maintained under conditions used for culture of blastocyst- and placenta-derived primary TS cells (5). Upon plating in trophoblast stem cell medium (TSCM) developed by Okae *et al*. (5), hESC-derived CTB-like cells underwent differentiation and epithelial colonies could not be retained after a single passage. CDX2 expression is upregulated significantly in as little as 2 days after initiation of hESC differentiation but decreases by day 6 (Fig. S1, *A* and *B*). In addition, previous studies have reported differentiation of hESCs to CDX2^+^/p63^+^ cells upon treatment with BMP for 4 days (30). Therefore, we explored the use of a shorter differentiation step for obtaining CTB-like cells (Fig. 3*A*). After 3 days of differentiation, H9 and H1 hESCs expressed nuclear CDX2, P63, and TEAD4 uniformly (Fig. 3*B*). However, by day 6 most differentiated H1 and H9 hESCs lose expression of CDX2 (Fig. 3*C*). Quantitative image analysis showed that nearly all cells are CDX2^+^ at day 3, in contrast to CTB-like cells at day 6. Of note, use of a 6-day protocol resulted in a significantly reduced fraction of CDX2^+^ cells in the case of H1 hESCs in comparison with the 3-day protocol; on the other hand, a significant fraction of H9 cells retained CDX2^+^ at day 6 (Fig. 3*D*). Transcriptome analysis using RNA sequencing identified 291 genes with significantly higher expression levels and 330 genes with significantly lower expression levels in day 3 differentiated hESCs versus undifferentiated hESCs (Tables S1 and S2).Expression of other trophectoderm-associated markers such as *HAND1*, *GATA3*, and *TFAP2A*, in addition to *CDX2*, was upregulated in differentiated hESCs at day 3, whereas expression of pluripotency-associated *NANOG* was downregulated. Gene set enrichment analysis of differentially expressed genes identified 567 and 202 gene ontology (GO) categories (of 9996 queried categories) associated with higher and lower gene expression in day 3 differentiated cells versus undifferentiated hESCs, respectively (Tables S3 and S4). Consistent with differentiation to epithelial trophoblast, genes associated with the GO terms for epithelium development, epithelial cell proliferation, and epithelial cell differentiation were upregulated in day 3 differentiated hESCs.

CDX2^+^ cells at day 3 were passaged into a chemically defined medium containing four major components (denoted TM4), the S1PR3 agonist CYM5541, the GSK3β inhibitor CHIR99021, the TGFβ inhibitor A83-01, and FGF10. CHIR99021 and A83-01 are components of TSCM used for culture of primary TS cells; FGF10 was included because FGFR2b signaling is active in blastocyst- and placenta-derived TS cells and the early placenta (5). Cells in TM4 could be maintained as epithelial colonies for 30+ passages over the course of 5 months. In TM4 medium, cells derived from H9 and H1 hESCs retained expression of the trophoblast markers CDX2, TFAP2C, YAP, TEAD4, and GATA3 (Figs. 3*E* and S6) (15, 17, 31, 32, 33, 34). In addition, cells expressed the pan-trophoblast marker KRT7 and low levels of P63. Of note, CDX2 expression has been strongly associated with the trophectoderm and is lost once placental villi are formed (30, 35, 36, 37). To indicate that these cells are derived from hPSCs, and to distinguish these cells from TS cells that do not express CDX2, these cells are denoted as hPSC-TS^CDX2^ cells.

We further evaluated the differentiation potential of hPSC-TS^CDX2^ cells using same protocols as those used by Okae *et al*. for differentiation of primary TS cells to EVTs and STB (5). Cells were able to form multinucleate STB that expressed hCG and KRT7 (Fig. 3*F*). However, upon EVT treatment, cells did not form mesenchymal elongated cells but acquired a flattened morphology. Upon passage, cells showed no HLA-G and minimal VE-Cadherin expression (Fig. 3*G*). Furthermore, cells maintained an epithelial flattened morphology with KRT7 expression but sparse hCG expression.

### CDX2^-^/P63^+^ TS cells derived from hESCs can be maintained in medium used for primary TS cells

**Figure 4:**
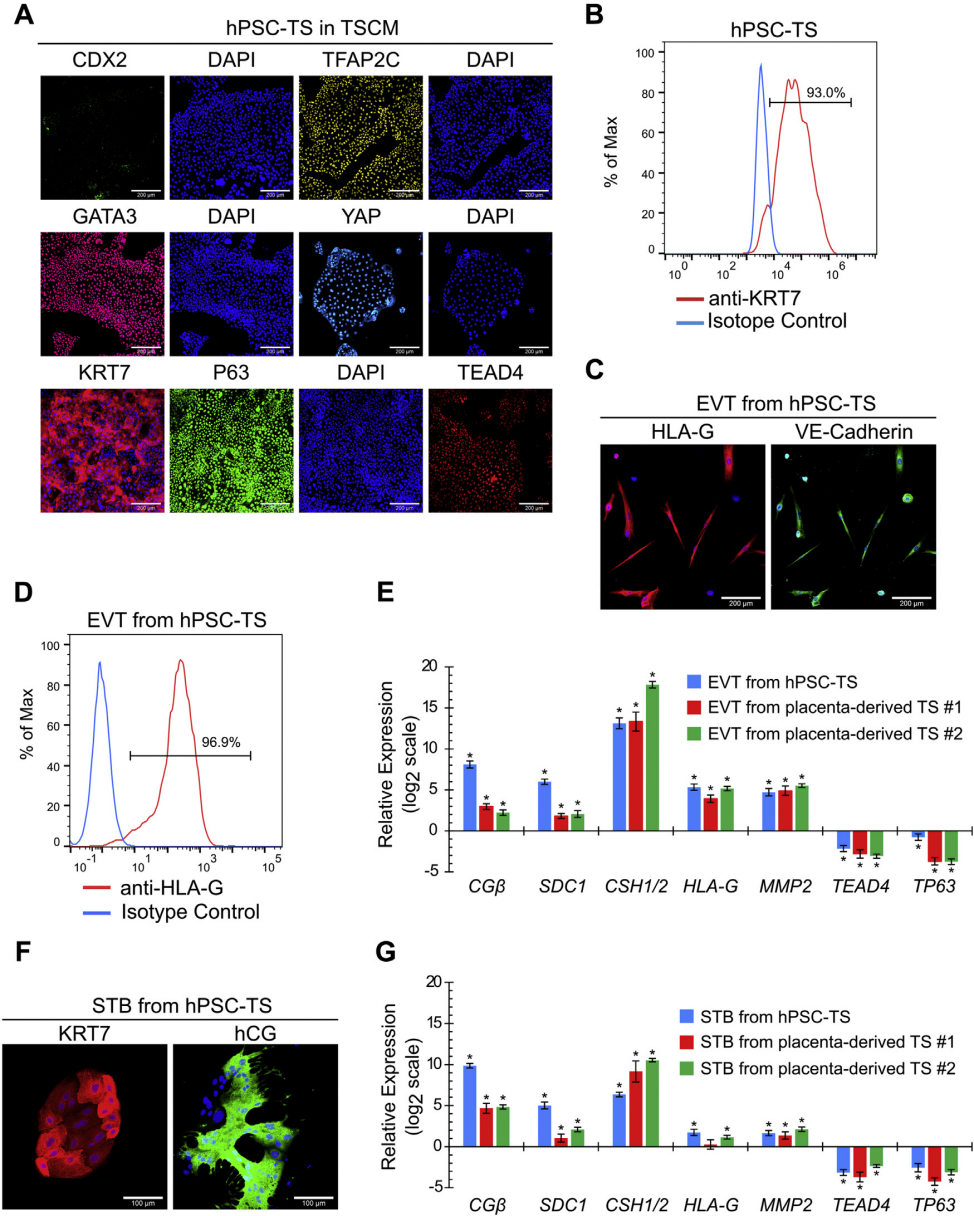
**Formation of hPSC-TS cells**. *A*, confocal images of H9 hPSC-TS in TSCM, staining for CDX2, TFAP2C, GATA3, YAP, TEAD4, and P63. Nuclei were stained with DAPI. The scale bars represent 200 μm. *B*, flow cytometry histogram of KRT7 expression of H9 hPSC-TS cells in TSCM compared with an isotype control. *C*, confocal images of EVTs from H9 hPSC-TS cells, staining for HLA-G and VE-Cadherin. Nuclei were stained with DAPI. The scale bars represent 200 μm. *D*, flow cytometry histogram of HLA-G expression of EVTs from H9 hPSC-TS cells compared with an isotype control. *E*, gene expression of *CGβ, SDC1, CSH1/2, HLA-G, MMP2, TEAD4*, and T*P63* of EVTs from H9 hPSC-TS- and placenta-derived TS #1 (CT30) and TS #2 (CT29) cells. Four biological replicates were used. (Error bars, SE, ∗*p* < 0.05 from TS cells). *F*, confocal images of STB from H9 hPSC-TS, staining for hCG and KRT7. Nuclei were stained with DAPI. The scale bars represent 100 μm. *G*, gene expression of *CGβ, SDC1, CSH1/2, HLA-G, MMP2, TEAD4*, and *TP63* of STB from H9 hPSC-TS and placenta-derived TS #1 (CT30) and TS #2 (CT29) cells. Four biological replicates were used. (Error bars, SE, ∗*p* < 0.05 from TS cells). DAPI, 4′,6-diamidino-2-phenylindole; EVT, extravillous trophoblast; hPSC, human pluripotent stem cell; STB, syncytiotrophoblast, TS, trophoblast stem; TSCM, trophoblast stem cell medium.

We evaluated whether hPSC-TS^CDX2^ cells could be maintained in TSCM used for culturing primary TS cells (Fig. 3*A*) (5). When hPSC-TS^CDX2^ cells cultured in TM4 for 5+ passages were directly passaged into TSCM, cells underwent a change in colony morphology over ∼3 passages; however, very little differentiation was observed. Of note, cell morphology of the hESC-derived cells closely resembled that of placenta-derived TS cells in TSCM that was used as a control (Fig. S7) (5). Strikingly, hPSC-TS^CDX2^ cells lost expression of CDX2 and gained higher expression of P63 in TSCM. As discussed earlier, cells could be maintained as epithelial colonies when hESCs after 3 days of differentiation were passaged into TM4. In contrast, passaging day 3 differentiated hESCs into TSCM resulted in extensive differentiation, although a few epithelial colonies could be observed. Further passaging resulted in similar morphological changes in the epithelial colonies as those observed for hPSC-TS^CDX2^ cells transitioning to TSCM. After ∼6 passages, only epithelial colonies remained, and they closely resembled both the hPSC-TS^CDX2^ cells transitioned into TSCM and placenta-derived TS cells. H9 and H1 hPSC-TS^CDX2^ cells, passaged directly into TSCM after 3 days of differentiation or transitioned from TM4 (Fig. 3*A*), showed high expression of YAP, TEAD4, TFAP2C, and GATA3, similar to cells in TM4, but no expression of CDX2 (Figs. 4*A* and S8*A*). Furthermore, they expressed the pan-CTB marker KRT7 (Fig. 4, *A* and *B*; Fig. S8, *A* and *B*). The hESC-derived cells cultured in TSCM exhibit a similar expression profile of trophoblast markers as placenta-derived TS cells (Fig. S7, *A* and *B*). Therefore, these cells are denoted as hPSC-TS cells to indicate that they are derived from hPSCs.

We further evaluated the differentiation potential of hPSC-TS cells using the same protocols as those used by Okae *et al*. for differentiation of primary TS cells to EVTs and STB (5). Similar to placenta-derived TS cell controls (Fig. S7, *C–E*), hPSC-TS cells could be differentiated into mesenchymal EVTs expressing HLA-G and VE-Cadherin (Fig. 4, *C* and *D*; Fig. S8, *C* and *D*), and multinucleate STB expressing hCG and KRT7 (Figs. 4*F* and S8*E*). In addition, the expression profile of transcripts corresponding to CTB, STB, and EVT markers upon differentiation of hPSC-TS cells was similar to those seen in the case of placenta-derived TS cell controls (Fig. 4, *E* and *G*). Furthermore, hPSC-TS cells have been maintained in TSCM for over 30 passages; they retain their ability to differentiate into STB and EVTs after long-term culture in TSCM. Taken together along with differences in culture conditions for maintenance, differentiation behavior, and expression of the trophectoderm marker CDX2, these results suggest that hPSC-TS^CDX2^ and hPSC-TS cells represent two distinct stem cell populations.

### Transcriptome analysis confirms high similarity between hPSC-TS cells and placenta-derived TS cells and reveals differences between hPSC-TS^CDX2^ and hPSC-TS cells

**Figure 5:**
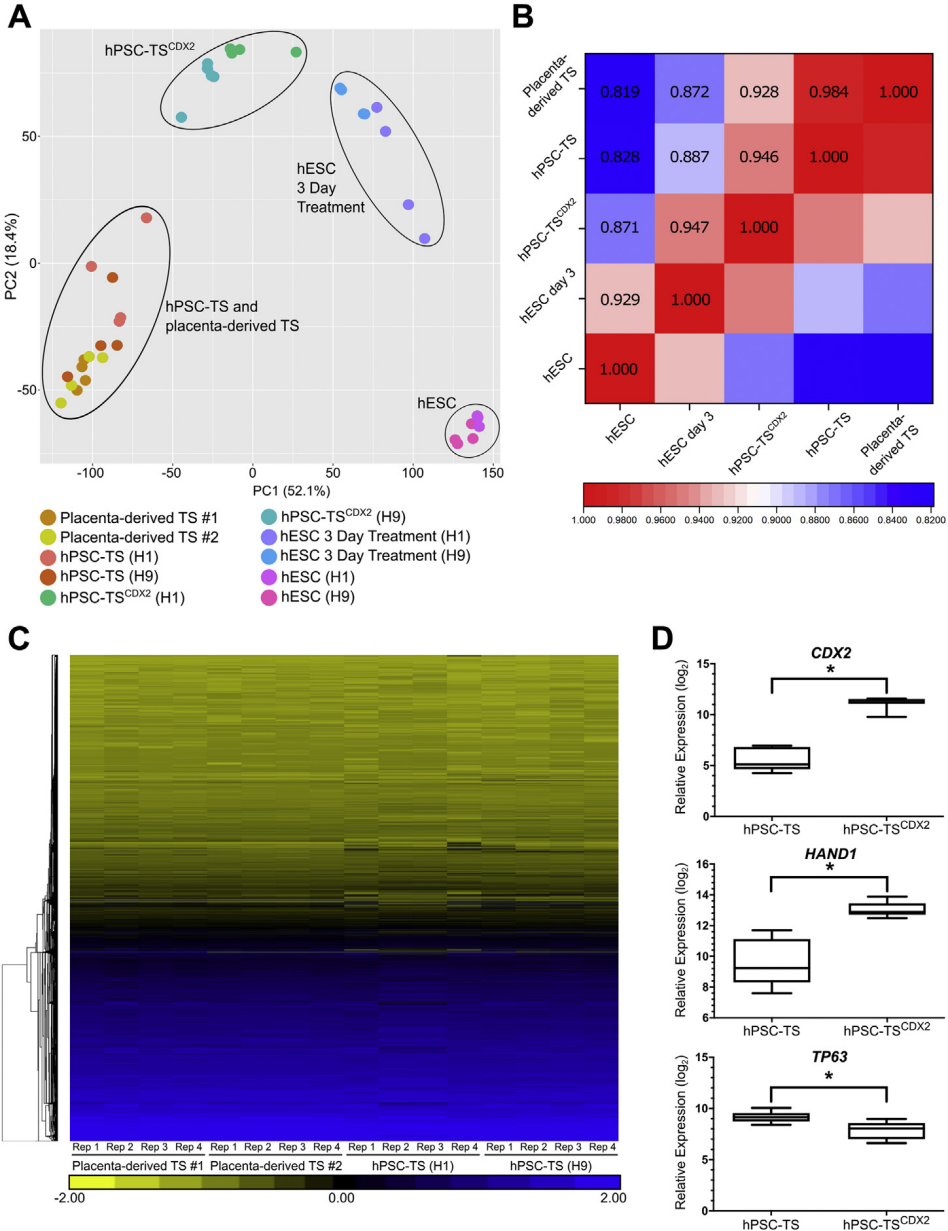
**Transcriptome analysis confirms equivalence of hESC-derived and primary TS cells and reveals differences between hPSC-TS and hPSC-TS**^**CDX2**^. *A*, principal component analysis of transcriptome data on H1 and H9 hESCs, H1 and H9 hESCs after 3 days treatment, H1 and H9 hESC-derived hPSC-TS^CDX2^ cultured in TM4, H1 and H9 hESC-derived hPSC-TS and placenta-derived TS #1 (CT30) and TS #2 (CT29) cultured in TSCM. *B*, spearman correlation coefficients for comparison between hESCs (H1 and H9), hESC after 3 days treatment (H1 and H9), hESC-derived hPSC-TS^CDX2^ cultured in TM4, hESC-derived hPSC-TS (H1 and H9) and placenta-derived TS #1 (CT30) and TS #2 (CT29) cultured in trophoblast stem cell medium (*p* < 0.00001). *C*, hierarchical clustering analysis of transcriptome data from H1 and H9 hESC-derived hPSC-TS and placenta-derived TS #1 (CT30) and TS #2 (CT29). Four biological replicates (*i.e.*, cells from different passages) were used. *D*, relative expression of trophectoderm-associated markers *CDX2* and *HAND1* and villous cytotrophoblast-associated marker *TP63* in hESC-derived hPSC-TS^CDX2^ and hPSC-TS (H1 and H9) (∗q < 0.001). hESC, human embryonic stem cell; hPSC, human pluripotent stem cell; TS, trophoblast stem.

We conducted genome-wide transcriptome analysis on hPSC-TS^CDX2^, hPSC-TS, and placenta-derived TS (control) cells using RNA sequencing. Note that, since hPSC-TS and placenta-derived TS cells are cultured under identical conditions, our analysis represents a direct comparison between transcriptome profiles across these two cell types. Principal component analysis (PCA) of transcriptomic signatures showed that hESC-derived and primary TS cells cluster together, indicating similarities in overall gene expression (Fig. 5*A*). A Spearman rank correlation test correlating average expression levels per gene between hPSC-TS and placenta-derived TS cells (Fig. 5*B*), and hierarchical clustering analysis (Fig. 5*C*), showed very high transcriptome similarity between hPSC-TS and placenta-derived TS cells. Note that 110 genes exhibit significant differential expression in hPSC-TS cells relative to placenta-derived TS cells (Table S5). In comparison, 109 genes show significant differentiation expression in hPSC-TS cells derived from H1 versus H9 hESCs (Table S6), underscoring the high transcriptome similarity between hPSC-TS and placenta-derived TS cells. Thus, in conjunction with similarities in marker expression and culture conditions for maintenance and differentiation, these results confirm that hPSC-TS are analogous to placenta-derived TS cells.

PCA also showed that hPSC-TS^CDX2^ cells are a distinct cell type that cluster differently from hPSC-TS cells and hESCs differentiated to the trophoblast lineage for 3 days (Fig. 5*A*). Statistical analysis of gene expression profiles identified genes that were significantly differentially expressed between hPSC-TS^CDX2^ and hPSC-TS. Specifically, 269 genes showed significantly higher expression levels and 275 genes showed significantly lower expression levels in hPSC-TS^CDX2^ versus hPSC-TS cells (Tables S7 and S8). Gene set enrichment analysis of these genes identified 300 and 47 GO categories (of 9996 queried categories) associated with genes showing higher and lower expression in hPSC-TS^CDX2^ versus hPSC-TS, respectively (Tables S9 and S10). Of interest, consistent with differences in colony morphology between hPSC-TS^CDX2^ and hPSC-TS cells, genes associated with extracellular matrix, biological adhesion, and cell-cell adhesion were upregulated in hPSC-TS^CDX2^ cells. Taken together along with distinct medium requirements for maintenance in cell culture, and differences in EVT differentiation under identical assay conditions, these results show that hPSC-TS and hPSC-TS^CDX2^ represent distinct stem cell populations.

Higher expression of the trophectoderm-associated markers *CDX2* and *HAND1* is observed in hPSC-TS^CDX2^ cells relative to hPSC-TS cells that are analogous to placenta-derived TS cells. On the other hand, expression of *TP63*, associated with villous CTB, is higher in hPSC-TS relative to hPSC-TS^CDX2^ (Fig. 5*D*). To investigate the similarities between the human trophectoderm and hPSC-TS^CDX2^ cells, we compared the transcriptome profiles of hPSC-TS^CDX2^, hPSC-TS, and placenta-derived TS cells with the transcriptome of trophectoderm cells from human embryos (38). The Spearman rank correlation test was used to correlate gene expression levels between primary trophectoderm cells and hPSC-TS^CDX2^, hPSC-TS, or placenta-derived TS cells (Table S11). The correlation R-values were similar for all three TS cell types and lower than those generating when comparing between hPSC-TS cells and placenta-derived TS cells or hPSC-TS^CDX2^ and hPSC-TS cells. The lower correlation R-values are likely due to the differences between cells in culture and primary human embryos and experimental protocols for transcriptome analysis; trophectoderm cells from human embryos were analyzed using single-cell RNA sequencing, as opposed to bulk RNA sequencing in our study. Additional studies are necessary to investigate whether hPSC-TS^CDX2^ cells are analogous to cells of the human trophectoderm.

### hPSC-TS^CDX2^ and hPSC-TS cells can be generated from hiPSCs

**Figure 6:**
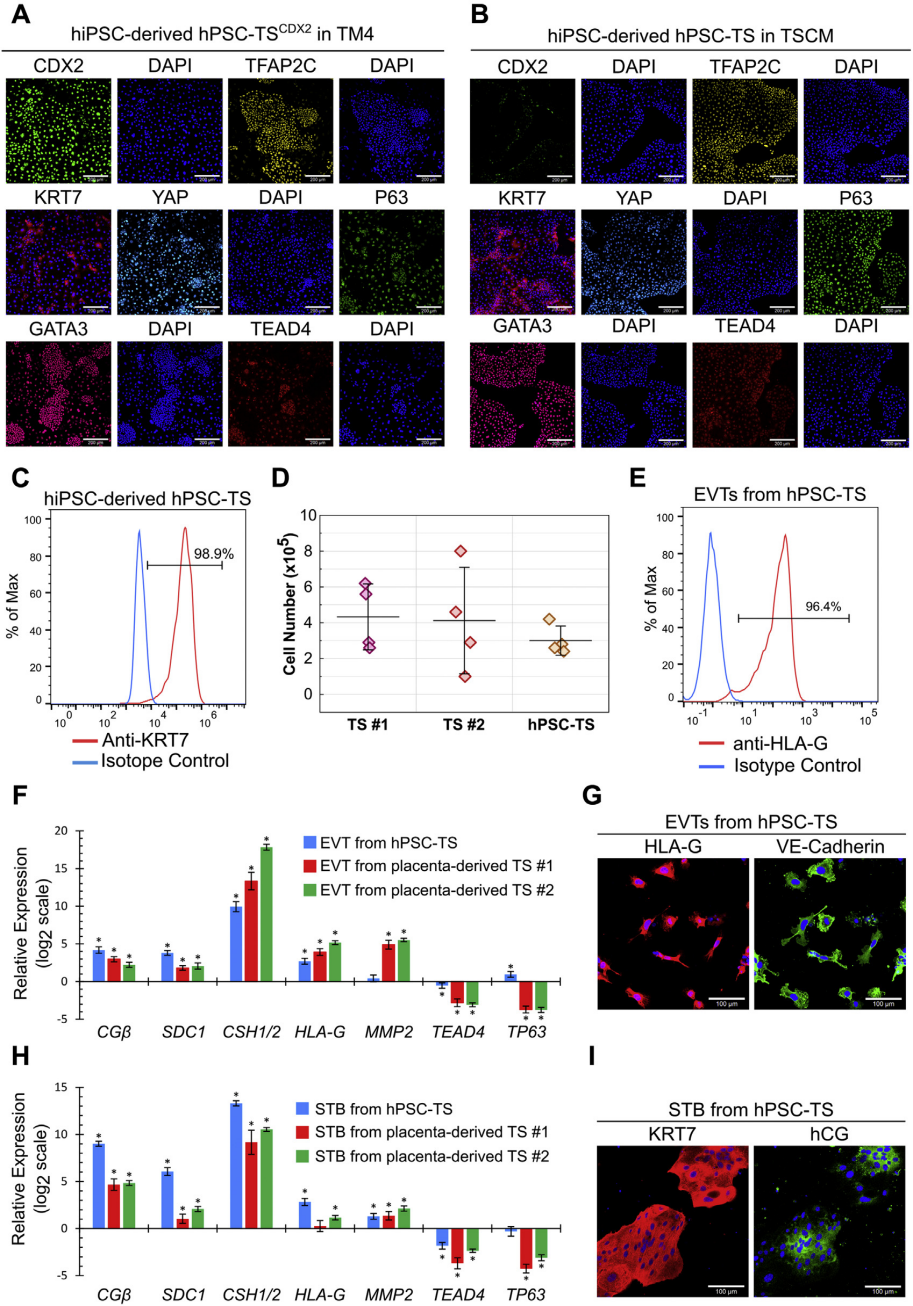
**hPSC-TS**^**CDX2**^**and hPSC-TS generated from hiPSCs**. *A*, confocal image of SC102A-1 hPSC-TS^CDX2^ in TM4, staining for CDX2, TFAP2C, GATA3, YAP, TEAD4, and P63. Nuclei were stained with DAPI. The *scale bars* represent 200 μm. *B*, confocal images of SC102A-1 hPSC-TS in TSCM, staining for CDX2, TFAP2C, GATA3, YAP, TEAD4, and P63. The scale bars represent 200 μm. *C*, flow cytometry histogram of KRT7 expression of SC102A-1 hPSC-TS cells in TSCM compared with an isotype control. *D*, proliferation of SC102A-1 hPSC-TS, placenta-derived TS #1 (CT30), and TS #2 (CT29) in TSCM. A total of 1 x 10^5^ cells were seeded and cells were counted after 3 days. Four biological replicates were used (error bars, SD). *E*, flow cytometry histogram of HLA-G expression of EVTs from SC102A-1 hPSC-TS cells compared with an isotype control. *F*, gene expression of *CGβ, SDC1, CSH1/2, HLA-G, MMP2, TEAD4*, and *TP63* of EVTs compared with TS cells from SC102A-1 hPSC-TS and placenta-derived TS #1 (CT30) and TS #2 (CT29). Four biological replicates were used (error bars, SE, ∗*p* < 0.05 for differential expression relative to TS cells). Data for placenta-derived TS cells is the same as used in Figure 4. *G*, confocal images of EVTs from SC102A-1 hPSC-TS, staining for HLA-G and VE-Cadherin. The scale bars represent 100 μm. *H*, gene expression of *CGβ, SDC1, CSH1/2, HLA-G, MMP2, TEAD4*, and *TP63* of STBs compared with TS cells from SC102A-1 hPSC-TS and placenta-derived TS #1 (CT30) and TS #2 (CT29). Four biological replicates were used (error bars, SE, ∗*p* < 0.05). *I*, confocal images of STB from SC102A-1 hPSC-TS, staining for hCG and KRT7. The scale bars represent 100 μm. EVT, extravillous trophoblast, hiPSC, human induced pluripotent stem cell; hPSC, human pluripotent stem cell; STB, syncytiotrophoblast; TS, trophoblast stem.

Finally, we investigated if our results on derivation of hPSC-TS^CDX2^ and hPSC-TS cells from hESCs could be extended to hiPSCs. Accordingly, we used our previously described protocols (Fig. 3*A*) to derive hPSC-TS^CDX2^ and hPSC-TS cells from the hiPSC line SC102A-1. hPSC-TS^CDX2^ cells derived from SC102A-1 hiPSCs maintained expression of CDX2, TFAP2C, GATA3, YAP KRT7, and TEAD4, along with a low expression level of P63 in TM4 medium (Fig. 6*A*). Similarly, hPSC-TS cells derived from SC102A-1 hiPSCs expressed KRT7, P63, TEAD4, TFAP2C, YAP, and GATA3 in TSCM (Fig. 6, *B* and *C*). SC102A-1 hPSC-TS cells lost expression of CDX2 but gained higher expression levels of P63 and KRT7 in TSCM. The proliferation rate of SC102A-1 hPSC-TS cells was also similar to that of placenta-derived TS cells (Fig. 6*D*). Differentiation of hPSC-TS cells derived from SC102A-1 hiPSCs using protocols described by Okae *et al*. (5) resulted in the formation of mesenchymal EVTs with high expression of HLA-G and VE-Cadherin (Fig. 6, *E* and *G*) and multinucleate STB expressing hCG and KRT7 (Fig. 6*I*). The expression profile of transcripts corresponding to CTB, STB, and EVT markers upon differentiation of SC102A-1 hPSC-TS cells was also similar to those seen in case of placenta-derived TS cell controls (Fig. 6, *F* and *H*). These results confirm that two distinct TS cell populations can also be derived from hiPSCs.

## Discussion

In this study, we have shown that two distinct stem cell populations of the trophectoderm lineage, hPSC-TS and hPSC-TS^CDX2^, can be derived from hESCs and hiPSCs under chemically defined culture conditions. Whether *bona fide* trophoblast can be obtained from hPSCs has been a subject of debate (7). Despite extensive research in this area, conducting a rigorous head-to-head comparison between hPSC-derived and primary trophoblasts has been challenging. The isolation of trophoblast stem cell populations from hPSCs in this study, in conjunction with the recent derivation of primary TS cells from blastocysts and early-gestation placental samples (5) enables such a comparison. We have shown that hPSCs can be differentiated to TS cells that express markers consistent with primary (placenta-derived) TS cells (P63, TEAD4, TFAP2C, YAP, and GATA3). The hPSC-derived hPSC-TS cells are cultured in the same medium as primary TS cells. They differentiate to EVT and STB using similar protocols as those used for primary TS cells. Furthermore, hPSC-derived hPSC-TS and primary TS have highly similar transcriptomes. Taken together, these results show that hPSC-derived TS cells are analogous to primary TS cells and that hPSCs can indeed differentiate to *bona fide* trophoblasts. In the time since our results were reported in preprint form (39), our approach for generation of TS cells from hPSCs has been independently confirmed; Shahbazi *et al*. used our protocol to generate hPSC-TS cells from hESCs overexpressing an E-Cadherin-GFP fusion (40). Our results are also consistent with other recent work that has demonstrated derivation of TS cells from hPSCs, although using different differentiation protocols and in the absence of BMP treatment (11). In these studies, hiPSCs were differentiated to trophoblast cysts in micromesh cultures for 30 to 50 days, and subsequently TS cells were obtained by culturing cells from cysts in TSCM.

### Comparison with other studies: role of specific culture conditions

Previous studies on trophoblast differentiation of hESCs have employed differing protocols, resulting in significantly different outcomes in some cases. Bernardo *et al*. reported that BMP treatment of hESCs results in differentiation of hESCs to mesoderm and not trophoblast (41). More recently, multiple studies have investigated the differentiation of hPSCs to the trophectoderm lineage. Dong *et al*. and Cinkornpumin *et al*. report the derivation of TS cells from naive hPSCs but not primed hPSCs (9, 10). In these studies, primed hPSCs did not give rise to TS cell lines when plated in TSCM that is used for maintenance of blastocyst- and placenta-derived TS cells in culture. Guo *et al*. claim that primed hPSCs do not undergo differentiation to the trophectoderm lineage using a previously described protocol involving treatment with BMP; rather they suggest that primed hPSCs differentiate to cells of the amnion (42). However, the differentiation protocols used in these studies differ significantly from that used in this study for deriving hPSC-TS cells from hPSCs. Specifically, our results show that receptor-mediated signaling by the albumin-associated sphingolipid S1P plays a critical role in hESC differentiation to trophoblast in our medium. Of note, receptor-mediated S1P signaling has been implicated in blastocyst formation in mouse (43). Differences in results reported by previous studies may be due to variability in the lipid composition of media used during trophoblast differentiation of hESCs.

Another possible explanation for discrepancies in previous studies is that differences in media used for routine maintenance of undifferentiated hPSCs may contribute to differences in differentiation potential. For instance, unlike hESCs cultured in the presence of KSR, hESCs in E8 medium exhibit some features of naive pluripotency (44).

Finally, further investigation is required to compare rigorously placenta-derived TS cells and cells of the human amniotic epithelium. It is important to note that we observe very high transcriptome similarity between hPSC-TS and placenta-derived TS cells (Fig. 5, *A*–*C*), and conversion of primed hPSCs to TS cells using our approach has been independently replicated (40).

### Differences between hPSC-TS^CDX2^ and hPSC-TS cells

Marker expression analysis, functional differentiation assays, and genome-wide transcriptome analysis confirm the high similarity between hPSC-TS and placenta-derived TS cells that are similar to villous CTB. However, hPSC-TS^CDX2^ cells differ significantly from hPSC-TS cells. They do not undergo differentiation to EVTs under the culture conditions used for differentiating hPSC-TS and primary TS cells. Moreover, transcriptome analysis shows that genes associated with several key pathways and biological processes are differentially regulated between hPSC-TS^CDX2^ and hPSC-TS cells. These results suggest that hPSC-TS^CDX2^ and hPSC-TS cells represent two distinct stem cell populations.

Significantly, hPSC-TS^CDX2^ cells, but not hPSC-TS, express high levels of the trophectoderm-associated markers *CDX2* and *HAND1*, associated with putative trophectoderm stem cells as proposed by Knöfler *et al*. (37). Furthermore, hPSC-TS^CDX2^ cells can be readily transitioned into TSCM used for culturing hPSC-TS, as was seen by Okae *et al*. (5) when transitioning trophectoderm cells of blastocysts into TSCM. Subsequently, hPSC-TS^CDX2^ lose expression of CDX2 and express higher levels of P63 in TSCM and can differentiate to form EVTs and STB. Note that TS cells derived from the trophectoderm in the blastocyst-stage embryo lose expression of CDX2 (5). On the other hand, it has not been possible yet to revert hPSC-TS to hPSC-TS^CDX2^ by culturing in TM4 medium. Finally, the trophectoderm forms a primitive STB and CTB upon implantation. Consistent with the trophectoderm, hPSC-TS^CDX2^ cells do not form EVT cells under the differentiation conditions used for EVT differentiation of hPSC-TS and placenta-derived TS cells. Taken together, these results raise the question whether hPSC-TS^CDX2^ cells may be a more primitive cell type than hPSC-TS cells, analogous to the human trophectoderm or simply a distinct cell type resulting from differences in cell culture conditions.

To investigate whether hPSC-TS^CDX2^ are similar to the human trophectoderm, we compared transcriptome data for hPSC-TS, hPSC-TS^CDX2^, and placenta-derived TS cells, with previously published data for trophectoderm cells from human embryos (38). However, our analysis showed greater similarity in average expression levels among different TS cell types in culture than between hPSC-TS^CDX2^ and primary trophectoderm cells; this is likely due to differences between cells in culture and primary human embryos and experimental protocols for transcriptome analysis. Furthermore, blastocyst-derived TS cell lines and some TS cell lines derived from naive human embryonic stem cells show defects in EVT differentiation (10). Therefore, further studies are needed to conclusively determine if hPSC-TS^CDX2^ cells are indeed similar to cells of the human trophectoderm and/or represent a more primitive trophoblast cell type than hPSC-TS cells.

### Considerations for derivation and culture of hPSC-TS^CDX2^ cells

To derive hPSC-TS^CDX2^ cells, undifferentiated hESCs maintained in E8 medium are first treated for 3 days with the S1PR3 agonist, BMP4, and the activin/nodal inhibitor SB4315432 to obtain CDX2^+^ cells. Subsequently, CDX2+ cells are passaged in TM4 medium to obtain hPSC-TS^CDX2^. Using this protocol, we observed increased differentiation of H1 hESC-derived cells upon passage into TM4 medium, relative to H9 hESC- and SC102A-1 hiPSC-derived cells. Shortening the initial treatment step in case of H1 hESCs to 2 days eliminated excessive differentiation and facilitated derivation of hPSC-TS^CDX2^ cells. However, we were unable to derive hPSC-TS^CDX2^ cells with hPSCs when the initial treatment was greater than 3 days.

It is important to note that hPSC-TS^CDX2^ cells proliferate slower in culture than hPSC-TS cells. They are passaged every 4 to 6 days at a 1:3 to 1:4 split ratio (as opposed to 1:4 to 1:6 for hPSC-TS cells). We also observe that the attachment of hPSC-TS^CDX2^ cells to tissue culture plates is less efficient than that of TS cells. Finally, we observe that excessive differentiation in TM4 medium during early passages could be countered by reducing the concentration of ascorbic acid (32 μg/ml instead of 64 μg/ml) in TM4. Additional studies on the composition of TM4 medium or the substrates used to coat tissue culture plates may lead to improved growth rate and attachment efficiency. Alternatively, the slower growth rate and less efficient attachment characteristics may be an inherent feature of the hPSC-TS^CDX2^ state. Nonetheless, we have successfully maintained hPSC-TS^CDX2^ derived from all cell lines studied for at least 20 passages, in several independent runs over 5+ months. We recommend passaging hPSC-TS^CDX2^ cells routinely at higher cell densities relative to hPSC-TS cells and troubleshooting cell line–specific variability by optimizing the initial treatment step and/or lowering ascorbic acid concentration in TM4.

### Derivation of hPSC-TS^CDX2^ and hPSC-TS cells from hiPSCs

We have shown that hPSC-TS^CDX2^ and hPSC-TS cells can be derived from hiPSCs. Since hiPSCs can be derived by reprogramming easily accessible somatic tissues, hPSC-TS and hPSC-TS^CDX2^ cells derived from hiPSCs can greatly accelerate research in placental biology. Furthermore, arguably a limitation of blastocyst- or placenta-derived hPSC-TS cells is that pregnancy outcomes at term for the early gestation placental samples or blastocyst stage embryos used cannot be predicted accurately. In contrast, hiPSC-derived hPSC-TS and hPSC-TS^CDX2^, from hiPSCs generated using somatic tissues obtained at term, will potentially enable development of models of validated normal and pathological trophoblast development. Pertinently, Sheridan *et al*. (6) have derived hiPSCs from umbilical cords of normal pregnancies and those associated with early-onset preeclampsia. Our results also gain particular significance in the light of restrictions on research with fetal tissue (45). However, although use of hiPSC-derived TS cells may shed light on the role of genetics in determining trophoblast pathology, reprogramming of somatic cells will likely alter their epigenome. Alteration of epigenetic signatures associated with placental pathology may limit the usefulness of these models. Thus, further research is needed to assess if TS cells derived from hiPSCs associated with placental pathology will retain disease phenotype.

In conclusion, using optimized cell culture protocols detailed in the current study, we have derived two distinct stem cell populations of the trophectoderm lineage—hPSC-TS^CDX2^ and hPSC-TS—from human pluripotent stem cells. These stem cell models will be powerful tools for *in vitro* studies on human trophoblast development.

## Experimental procedures

### Key resources

Key resources used in this study are listed in Table 1.

**Table 1:** Key resources

Reagent or resource	Source	Identifier
hPSC cell lines		
H1 hESCs	Wicell	RRID: CVCL_9771
H9 hESCs	Wicell	RRID: CVCL_9773
SC102A-1 hiPSCs	Systems Biosciences	RRID: CVCL_IT66
Antibodies and staining reagents		
Anti-KRT7	Santa Cruz Biotechnology	Cat#sc-23876, RRID:AB_2265604
Anti-KRT7	Cell Signaling Technologies	Cat# 4465, RRID:AB_11178382
Anti-hCG	Abcam	Cat# ab9582, RRID:AB_296507
Anti-hCG	Abcam	Cat# ab9376, RRID:AB_307221
Anti-P63	Cell Signaling Technologies	Cat# 13,109, RRID:AB_2637091
Anti-GATA3	Cell Signaling Technologies	Cat# 5852, RRID:AB_10835690
Anti-TFAP2C	Cell Signaling Technologies	Cat# 2320, RRID:AB_2202287
Anti-YAP	Cell Signaling Technologies	Cat# 4912, RRID:AB_2218911
Anti-TEAD4	Abcam	Cat# ab58310, RRID:AB_945789
Anti-CDX2	Abcam	Cat# ab76541, RRID:AB_1523334
Anti-VE-Cadherin	Cell Signaling Technologies	Cat# 2500, RRID:AB_10839118
Anti-HLA-G	Abcam	Cat# ab52455, RRID:AB_880552
Anti-Syncytin	Santa Cruz Biotechnology	Cat# sc-50369, RRID:AB_2101536
Rabbit Polyclonal IgG	R&D Systems	Cat# AB-105-C, RRID:AB_354266
Rabbit XP IgG	Cell Signaling Technologies	Cat# 3900, RRID:AB_1550038
Mouse IgG1	Abcam	Cat# ab18447, RRID:AB_2722536
Mouse IgG2a	Abcam	Cat# 554126, RRID:AB_479661
Alexa Fluor 488–conjugated anti-rabbit IgG	Thermo Fisher Scientific	Cat# A-11034, RRID:AB_2576217
Alexa Fluor 647–conjugated anti-rabbit IgG	Thermo Fisher Scientific	Cat# A-21052, RRID:AB_2535719
DAPI	R&D Systems	Cat#5748
CellMask deep red plasma membrane stain	Invitrogen	Cat#C10046
Chemicals, Peptides, and Recombinant Proteins		
TrypLE	Thermo Fisher Scientific	Cat#12604013
Vitronectin	Thermo Fisher Scientific	Cat#A14700
Laminin 521	Stem Cell Technologies	Cat#77003
Human FGF-10	Stem Cell Technologies	Cat#78037
TeSR-E8	Stem Cell Technologies	Cat#05990
TeSR-E7	Stem Cell Technologies	Cat#05914
TeSR-E6	Stem Cell Technologies	Cat#05946
ReLeSR	Stem Cell Technologies	Cat#05872
Sphingosine-1-phosphate	Tocris	Cat#1370
D-erythro-dihydrosphingosine-1-phosphate	Abcam	Cat#ab141750
SB431542	Tocris	Cat#1614
BMP4	Thermo Fisher Scientific	Cat#PHC9534
CYM5442 hydrochloride	Tocris	Cat#3601
CYM5520	Tocris	Cat#5418
CYM5541	Tocris	Cat#4897
Y-27632 dihydrochloride	Tocris	Cat#1254
EGF	R&D Systems	Cat#236-EG
Doxycycline hyclate	Tocris	Cat#4090
Puromycin dihydrochloride	Tocris	Cat#4089
Activin A	R&D Systems	Cat#338-AC
Greiner Bio-one Cell View glass plates	Greiner Bio-one	Cat#627965
4% Paraformaldehyde in PBS	Thermo Fisher Scientific	Cat#R37814
Triton X-100	Sigma	Cat#T8787
PBS w/o CaMg	Sigma	Cat#D5773
PBS w/CaMg	Sigma	Cat#D8662
Human IgG	Immunoreagents	Cat#Hu-003-C
BSA	Fisher Scientific	Cat#BP9703
10% BSA fatty acid free in PBS	Sigma	Cat#A1595
VPA	Sigma	Cat#P6273
A83–01	Tocris	Cat#2939
2-mercaptoethanol	Sigma	Cat#M3148
FBS	Thermo Fisher Scientific	Cat#16141–061
DMEM/F12	Thermo Fisher Scientific	Cat#11320033
ITS-X	Thermo Fisher Scientific	Cat#51500–056
L-ascorbic acid	Sigma	Cat#A8960
Pen/Strep	Thermo Fisher Scientific	Cat#15140122
Forskolin	Tocris	Cat#1099
Neuregulin	Cell Signaling Technologies	Cat#5218SC
Matrigel	Corning	Cat#354234
KSR	Thermo Fisher Scientific	Cat#10828028
Trizol Reagent	Thermo Fisher Scientific	Cat#15596018
DEPC	Sigma	Cat#95284
Baseline Zero DNAase Kit	VWR	Cat#76081–624
Oligo-dT	IDT	Cat#51–01–15–07
dNTP mix	Thermo Fisher Scientific	Cat#10297018
Superscript II RT	Thermo Fisher Scientific	Cat#18064014
SYBR Green Supermix	Bio-rad	Cat#1725272
Methanol	Fisher Scientific	Cat#A412–500
Acetone	Fisher Scientific	Cat#A18–500
Critical Commercial Kits		
GeneJET RNA Purification Kit	Thermo Fisher Scientific	Cat#K0731
Oligonucleotides		
qPCR Primers	IDT	Methods S1 for primer sequences
Software and Algorithms		
R (v3.6.0)	http://www.R-project.org/	N/A
DESeq2 package (v1.22.2)		
PCR package (v1.2.2)		
SAS Software		N/A
Zeiss Zen Software	https://www.zeiss.com/microscopy/us/products/microscope-software/zen-lite.html	N/A

### Culture of hPSCs

H1 and H9 hESCs and SC102A-1 hiPSCs were cultured on plates coated with vitronectin (5 μg/ml) at room temperature for at least 1 h. Cells were cultured in 2 ml of TeSR-E8 medium at 37 °C in 5% CO_2_ in 6-well plates, and the culture medium was replaced every day. When cells reached confluency, they were passaged using ReLeSR according to the manufacturer’s protocol, at a 1:10 split ratio.

### Differentiation of hPSCs (6-day protocol)

The day after passaging, differentiation was initiated in hPSCs by treatment with S1P (10 μM), SB431542 (25 μM), and BMP4 (20 ng/ml) in TeSR-E7 for 6 days. In some experiments, the S1PR agonists CYM5442 hydrochloride (10 nM), CYM5520 (5 μM), or CYM5541 (2 μM) were added during the differentiation process. The medium was replaced every day. At day 6 of treatment, cells were dissociated with TrypLE for 5 min at 37 °C. For differentiation to EVTs, cells were seeded in a 6-well plate precoated with 5 μg/ml of vitronectin at a density of 7 × 10^4^ cells per well and cultured in 2 ml of EVT medium (TeSR-E8 medium supplemented with SB431542 [25 μM] and EGF [2.5 ng/ml]). The medium was replaced every other day and analyzed at day 12 of total treatment. For differentiation to STB, cells were seeded in a 6-well plate precoated with 5 μg/ml of vitronectin at a density of 4 × 10^4^ cells per well and cultured in 2 ml of STB medium (TeSR-E6 supplemented with Activin A [20 ng/ml] and EGF [50 ng/ml]). The medium was replaced every other day and analyzed at day 14 of total treatment.

### Differentiation of hPSCs to hPSC-TS^CDX2^ and hPSC-TS cells

The day after passaging, hPSCs were differentiated by treatment with CYM5541 (2 μM), SB431542 (25 μM), and BMP4 (20 ng/ml) in TeSR-E7 for 2 and 3 days for H1 and H9 hESCs, respectively. The medium was replaced every day. After 2 or 3 days of treatment, cells were dissociated with TrypLE for 5 min at 37 °C. For propagation of hPSC-TS^CDX2^ cells, all cells were seeded in a 6-well plate precoated with 3 μg/ml of vitronectin and 1 μg/ml of Laminin 521 at a density of ∼5 × 10^4^ cells per well and cultured in 2 ml of TM4 medium (TeSR-E6 medium supplemented with CYM5541 [2 μM], A 83-01 [0.5 μM], FGF10 [25 ng/ml], and CHIR99021 [2 μM]). For establishment of hPSC-TS cells, all cells were seeded in a 6-well plate precoated with 3 μg/ml of vitronectin and 1 μg/ml of Laminin 521 at a density of ∼5 × 10^4^ cells per well and cultured in 2 ml of TSCM developed by Okae *et al*. (Dulbecco's modified Eagle's medium [DMEM]/F12 supplemented with 0.1 mM 2-mercaptoethanol, 0.2% fetal bovine serum, 0.5% Penicillin-Streptomycin, 0.3% BSA, 1% ITS-X supplement, 1.5 μg/ml L-ascorbic acid, 50 ng/ml EGF, 2 μM CHIR99021, 0.5 μM A83-01, 1 μM SB431542, 0.8 mM VPA, and 5 μM Y27632) (5). hPSC-TS^CDX2^ cells were directly passaged into TSCM for formation of hPSC-TS cells; complete transition took ∼5 passages. Alternatively, hPSCs after 2 or 3 days of differentiation were directly passaged into TSCM.

### Culture of hPSC-TS^CDX2^ and hPSC-TS cells

hPSC-TS^CDX2^ and hPSC-TS cells were cultured in TM4 and TSCM, respectively, in 2 ml of culture medium at 37 °C in 5% CO_2_. Culture medium was replaced every 2 days. When hPSC-TS^CDX2^ and hPSC-TS cells reached 70% to 90% confluence, they were dissociated with TrypLE at 37 °C for 5 to 10 min and passaged to a new 6-well plate precoated with 3 μg/ml of vitronectin and 1 μg/ml of Laminin 521 at a 1:3 to 1:4 split ratio for hPSC-TS^CDX2^ and 1:4 to 1:6 split ratio for hPSC-TS cells. hPSC-TS^CDX2^cells grown in TM4 medium were supplemented with Y-27632 upon passage to aid in single cell attachment. Cells were routinely passaged approximately every 4 to 6 days. hPSC-TS^CDX2^ and hPSC-TS cells at passages 5+ were used for analysis, with the exception of one replicate of H1-derived hPSC-TS^CDX2^ used in RNA sequencing analysis where cells at passage 2 in TM4 were used.

Placenta-derived TS cells, CT30 (female) and CT29 (male), a kind gift from Drs Hiroaki Okae and Takahiro Arima (Tohoku University, (5)),- were grown and passaged the same way in TSCM as hPSC-TS cells.

### Differentiation of hPSC-TS^CDX2^ and hPSC-TS cells

hPSC-TS cells were grown to ∼80% to 90% confluence in TSCM and dissociated with TrypLE for 10 min at 37 °C. For differentiation to EVTs and STB, slightly modified versions of protocols developed by Okae *et al*. were used (5). For differentiation to EVTs, hPSC-TS cells were seeded in 6-well plates precoated with 3 μg/ml vitronectin and 1 μg/ml of Laminin 521 at a density of 1.25 × 10^5^ cells per well and cultured in 2 ml of EVT medium (DMEM/F12 supplemented with 0.1 mM 2-mercaptoethanol, 0.5% Penicillin-Streptomycin, 0.3% BSA, 1% ITS-X supplement, 100 ng/ml NRG1, 7.5 μM A83-01, 2.5 μM Y27632, and 4% KSR). Matrigel was added to a final media concentration of 2% after suspending the cells in EVT medium. At day 3, the medium was replaced with the EVT medium without NRG1 and Matrigel was added to a final concentration of 0.5%. At day 6, cells were dissociated with TrypLE for 15 min at 37 °C and passaged to new vitronectin/laminin-coated 6-well plates at a 1:2 split ratio. The cells were suspended in the EVT medium without NRG1 and KSR. Matrigel was added to a final concentration of 0.5%, and cells were analyzed after two additional days of culturing. For differentiation to STB, cells were seeded in 6-well plates precoated with 3 μg/ml vitronectin and 1 μg/ml of Laminin 521 at a density of 1.5 × 10^5^ cells per well and cultured in 2 ml of DMEM/F12 supplemented with 0.1 mM 2-mercaptoethanol, 0.5% Penicillin-Streptomycin, 0.3% BSA, 1% ITS-X supplement, 2.5 μM Y27632, 2 μM forskolin, and 4% KSR. The medium was replaced at day 3, and cells were analyzed at day 6.

### RNA isolation, cDNA synthesis, and quantitative PCR

RNA was isolated using Trizol reagent using the manufacturer’s protocol. For cDNA synthesis, the RNA pellet was dissolved in diethyl pyrocarbonate (DEPC)-treated water. The RNA was purified using Baseline-ZERO DNase buffer and Baseline-ZERO DNase enzyme and incubated at 37 °C for 30 min. The purification was stopped with Baseline-ZERO DNase stop solution and heated at 65 °C for 10 min. cDNA was synthesized using 18-mer Oligo-dT and dNTP mix and heated to 65 °C for 5 min and quickly chilled on ice. First strand buffer and DTT was added and incubated at 42 °C for 2 min, then superscript II RT enzyme was added and incubated at 42 °C for 50 min. The enzyme was inactivated at 70 °C for 15 min. The cDNA was stored at −20 °C until further used. The quantitative PCR (qPCR) reaction was carried out using SYBR Green Supermix in a C1000 Touch Thermal Cycler CFX384 Real-Time System (Bio-Rad). The primers used for qPCR analysis are listed in Methods S1. ANOVA analysis of gene expression data was carried out with SAS and package PCR in R software using the ΔΔCt method to determine gene expression changes (46). qPCR analysis was carried out using at least three biological replicates.

### Immunofluorescence analysis

For immunofluorescence analysis, cells were grown on glass-bottom culture dishes coated with 3 μg/ml vitronectin and 1 μg/ml of Laminin 521. Cells were fixed using 4% paraformaldehyde in PBS for 10 min, permeabilized with 0.5% Triton X-100 for 5 min, and blocked in 3% BSA/PBS with 0.1% human IgG and 0.3% Triton X-100 for 1 h. Cells were then incubated overnight with the primary antibody diluted in blocking buffer. The following primary antibodies were used: anti-KRT7 (SCB, 1:50), anti-KRT7 (CST, 1:500), rabbit anti-hCG (1:100), mouse anti-hCG (1:100), anti-YAP (1:200), anti-TFAP2C (1:400), anti-P63 (1:600), anti-GATA3 (1:500), anti-TEAD4 (1:250), anti-CDX2 (1:300), anti-VE-Cadherin (1:400), anti-HLA-G (1:300), anti-syncytin (1:50). Corresponding isotype controls (rabbit polyclonal IgG, rabbit XP IgG, mouse IgG1, and mouse IgG2a) were used at primary antibody concentrations. Alexa Fluor 488– or Alexa Fluor 647–conjugated secondary antibodies were used as secondary antibodies. Nuclei were stained with DAPI, and all samples were imaged using a Zeiss LSM 710 or 880 laser scanning confocal microscope (Carl Zeiss).

### Confocal image analysis

Image analysis was conducted using an image processing algorithm created in MATLAB. First, the DAPI stain was isolated, binarized, and processed to accurately represent the number of cells in each image. The primary antibody stain of interest was isolated and processed in the same manner. Only primary antibody pixels that overlap DAPI pixels were considered for analysis, and the average intensities of those pixels were measured and correlated to the nearest nuclei. This was performed for one control image and multiple experimental images. Each cell in the experimental images was considered positively stained if the average intensity of that cell was greater than the average intensity of all cells in the control image. The average intensity of cells in the control image was subtracted from the average intensity for each individual cell across all images for each experimental condition to eliminate background. If no fluorescence signal was detected or if the average intensity was below the average intensity of the control image, then the expression for that cell was set to zero. The fraction of cells expressing a specific protein in each image was calculated as the ratio of the number of cells with non-zero fluorescence intensity to the total number of cells. Statistical analysis was done using a two-tailed *t*-test evaluating percent positive cells from different treatment groups.

### Flow cytometry analysis

For flow cytometry analysis, cells were dissociated with TrypLE for 5 min at 37 °C. Cells were fixed in suspension in 2% paraformaldehyde in PBS for 5 min at room temperature. Cells were permeabilized and blocked in 1% BSA/PBS with 1 mg/ml Saponin (Sigma 47036-50G-F) for 15 min at room temperature. Cells were then incubated for 1 h on ice with the primary antibody diluted in the blocking buffer. The corresponding isotype control was used at the primary antibody concentration. Subsequently, cells were incubated in an Alexa Fluor 488–conjugated secondary antibody on ice protected from light for 1 h and analyzed immediately in a 1% BSA/PBS buffer. A BD Accuri C6 Plus Flow Cytometer was used for analysis. Data from 10,000 events were collected.

### RNA sequencing analysis using next-generation sequencing

Total RNA was extracted with Trizol reagent using manufacturer’s protocol. RNA from four biological replicates (*i.e.*, cells from different passages) for cell line/type assessed was purified using GeneJET RNA Purification Kit using manufacturer’s protocol. Isolated RNA samples were then used to evaluate genome-wide mRNA expression profiles using next-generation RNA sequencing, conducted at GENEWIZ, LLC. RNA samples received at GENEWIZ were quantified using Qubit 2.0 Fluorometer (Life Technologies), and RNA integrity was checked using Agilent TapeStation 4200 (Agilent Technologies).

RNA sequencing libraries were prepared using the NEBNext Ultra RNA Library Prep Kit for Illumina following manufacturer’s instructions (NEB). Briefly, mRNAs were first enriched with Oligo(dT) beads. Enriched mRNAs were fragmented for 15 min at 94 °C. First strand and second strand cDNAs were subsequently synthesized. cDNA fragments were end repaired and adenylated at 3′ends, and universal adapters were ligated to cDNA fragments, followed by index addition and library enrichment by limited-cycle PCR. The sequencing libraries were validated on the Agilent TapeStation (Agilent Technologies) and quantified by using Qubit 2.0 Fluorometer (Invitrogen) as well as by quantitative PCR (KAPA Biosystems).

The sequencing libraries were clustered on four lanes of a flowcell. After clustering, the flowcell was loaded on the Illumina HiSeq 4000 instrument according to manufacturer’s instructions. The samples were sequenced using a 2 x 150 bp Paired End (PE) configuration. Image analysis and base calling were conducted by the HiSeq Control Software. Raw sequence data (.bcl files) generated from Illumina HiSeq were converted into fastq files and de-multiplexed using Illumina's bcl2fastq 2.17 software. One mismatch was allowed for index sequence identification.

After investigating the quality of the raw data, sequence reads were trimmed to remove possible adapter sequences and nucleotides with poor quality using Trimmomatic v.0.36. The trimmed reads were mapped to the *Homo sapiens* GRCh38 reference genome available on ENSEMBL using the STAR aligner v.2.5.2b. The STAR aligner is a splice aligner that detects splice junctions and incorporates them to help align the entire read sequences. BAM files were generated as a result of this step. Unique gene hit counts were calculated by using feature Counts from the Subread package v.1.5.2. Only unique reads that fell within exon regions were counted.

### Analysis of gene expression profiles

After extraction of gene hit counts, the gene hit counts table was used for downstream differential expression analysis. Genome-wide RNA sequencing count data were processed and statistically assessed using the DESeq2 package (v1.22.2) in R Software (3.6.0) (https://www.r-project.org/). Count data were first filtered to include transcripts expressed above background, requiring the median across samples to be greater than the overall median signal intensity, as implemented in DESeq2. Count data were then normalized by median signal intensity using algorithms enabled within DESeq2, resulting in variance stabilized expression values (47). These normalized values were used to carry out a PCA comparing data-reduced global expression signatures across samples. Principal components were calculated and visualized using the prcomp function in R (https://www.rdocumentation.org/packages/stats/versions/3.6.2/topics/prcomp). The average gene expression levels of different cell types were compared using the Spearman rank correlation test. Transcriptome profiles obtained by single-cell RNA sequencing of human embryos, and annotated as trophectoderm (38), were combined for comparison with gene expression data from human trophectoderm cells. Heat maps were generated using Partek Genomics Suite Software (v7.18.0723) and gene-specific plots using GraphPad Prism Software (v8.2.0), based on normalized expression values.

### Statistical and gene set enrichment analysis of differentially expressed genes

Genes that showed the greatest difference in expression between the day 3 differentiated hESCs and undifferentiated hESCs, and hPSC-TS^CDX2^ and hPSC-TS cells were identified using an analysis of variance analysis (ANOVA) comparing the normalized expression levels between these two groups. Genes showing the greatest difference in expression between hPSC-TS^CDX2^ and hPSC-TS cells were identified using the following statistical filters: (1) a false discovery rate–corrected q-value<0.05 (48) and (2) a fold change in expression (ratio of average across hPSC-TS^CDX2^ and hPSC-TS cells samples) ≥ ± 1.5. To evaluate the biological role of these genes, a gene set enrichment analysis was carried out on the genes identified as significantly differentially expressed between groups. Specifically, all GO gene sets (n = 9996) from the Molecular Signature Database (MSigDB) (http://www.gsea-msigdb.org/gsea/msigdb/index.jsp) were queried for using the right-tailed Fisher’s Exact test, as enabled through the “platform for integrative analysis of omics data” (PIANO) packing in R (49). Gene sets were required to have an enrichment *p*-value<0.01 to be considered significant, consistent with previously published methods (50, 51). Genes that were identified at higher expression levels were evaluated separately from genes identified at significantly lower expression levels in day 3 differentiated hESCs versus undifferentiated hESCs, and hPSC-TS^CDX2^ versus hPSC-TS cells.

## Data availability

RNA sequencing data associated with this study have been deposited in Gene Expression Omnibus (GEO; accession number GSE137295). All other data that support the findings of this study are available within the article and its supplementary materials.

## Supporting information

This article contains supporting information.

## Conflict of interest

The authors declare that they have no conflicts of interest with the contents of this article.
