# Supplementary material for: Two distinct trophectoderm lineage stem cells from human pluripotent stem cells
Source: J Biol Chem. 2021 Feb 5;296:100386. doi: 10.1016/j.jbc.2021.100386 (PMC7948510; doi:10.1016/j.jbc.2021.100386)
Supplement: Figures S1–S8 [file mmc1.pdf]

Supplemental Information

Two distinct trophectoderm lineage stem cells from human pluripotent stem cells

Adam Mischler[1], Victoria Karakis[1], Jessica Mahinthakumar[1], Celeste K. Carberry[2], Adriana San Miguel[1], Julia E. Rager[2], Rebecca C. Fry[2], Balaji M. Rao[1,3*]

[1]Department of Chemical and Biomolecular Engineering, North Carolina State University, Raleigh, North Carolina 27695, USA

[2]Department of Environmental Sciences and Engineering, Institute for Environmental Health Solutions, University of North Carolina - Chapel Hill, Chapel Hill, North Carolina 27599, USA

[3]Golden LEAF Biomanufacturing Training and Education Center (BTEC), North Carolina State University, Raleigh, North Carolina 27695, USA

*Corresponding author: Email: bmrao@ncsu.edu

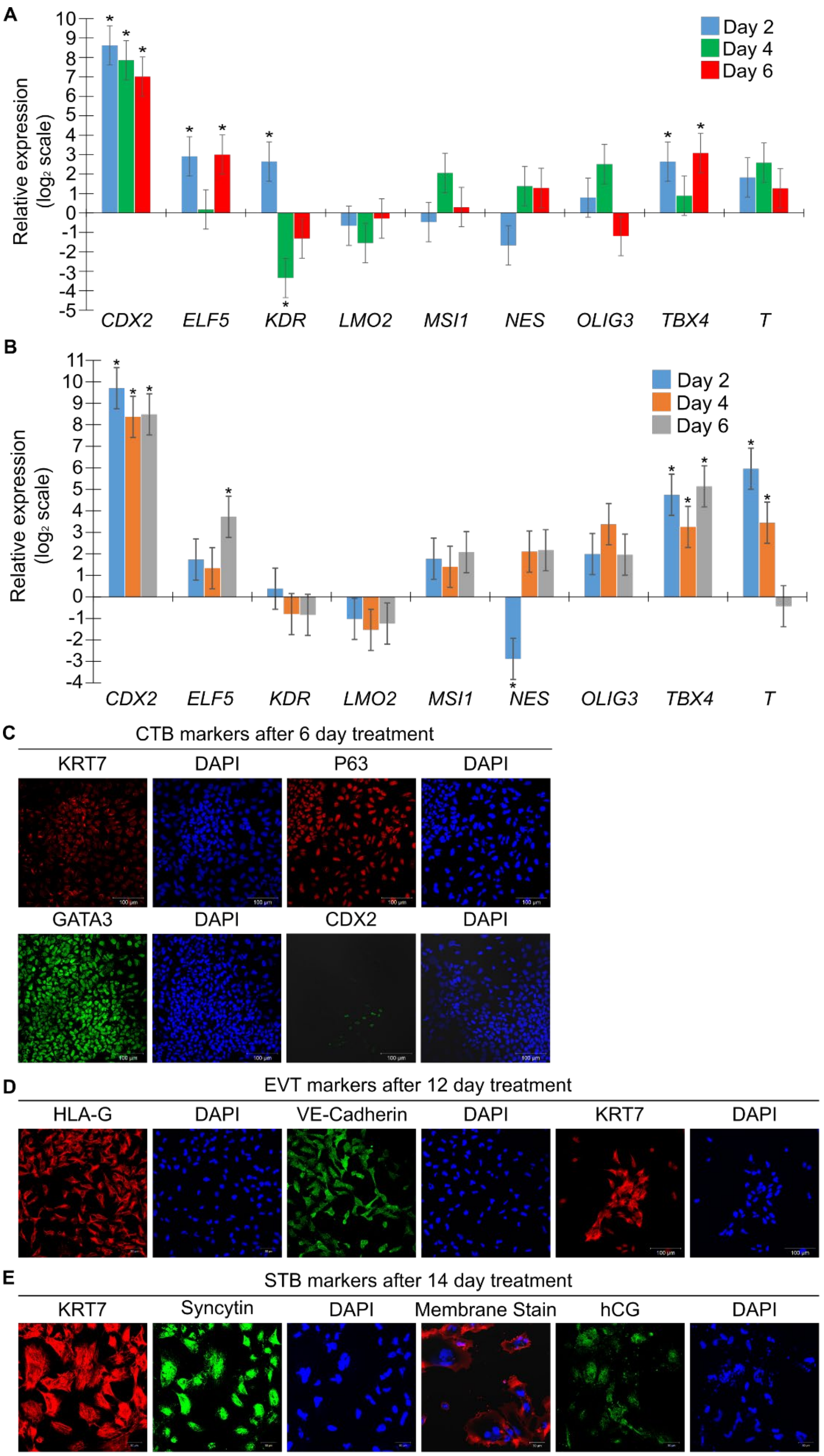
A
Relative expression (log2 scale)
Day 2
Day 4
Day 6
CDX2
ELF5
KDR
LMO2
MSI1
NES
OLIG3
TBX4
T
B
Relative expression (log2 scale)
Day 2
Day 4
Day 6
CDX2
ELF5
KDR
LMO2
MSI1
NES
OLIG3
TBX4
T
C
CTB markers after 6 day treatment
KRT7
DAPI
P63
DAPI
GATA3
DAPI
CDX2
DAPI
D
EVT markers after 12 day treatment
HLA-G
DAPI
VE-Cadherin
DAPI
KRT7
DAPI
E
STB markers after 14 day treatment
KRT7
Syncytin
DAPI
Membrane Stain
hCG
DAPI

Figure S1: A chemically defined medium containing S1P enables differentiation of hESCs to CTB and terminally differentiated trophoblasts. Related to Figure 1.

(A) Gene expression of CTB: *CDX2, ELF5;* Mesoderm: *KDR, LMO2, T* (brachyury)*, TBX4* and neural genes*: MSH1, NES* (nestin), *OLIG3* in H9 hESCs undergoing differentiation, compared to hESCs. Three biological replicates were used. (Error bars, S.E., $^*p<0.05$).

(B) Gene expression of CTB: *CDX2, ELF5;* Mesoderm: *KDR, LMO2, T* (brachyury)*, TBX4* and neural genes*: MSH1, NES* (nestin), *OLIG3* in H1 hESCs undergoing differentiation compared to hESCs. Three biological replicates were used. (Error bars, S.E., $^*p<0.05$).

(C) Confocal images of CTB from 6-day initial treatment of H1 hESCs, staining for KRT7, P63, GATA3, and CDX2. Nuclei were stained with DAPI.

(D) Confocal images of EVTs from 12-day treatment of H1 hESCs, staining for KRT7, HLA-G and VE-Cadherin. Nuclei were stained with DAPI.

(E) Confocal images of STB from 14-day treatment of H1 hESCs, staining for KRT7, syncytin and hCG. Nuclei were stained with DAPI. Membrane was stained with CellMask deep red plasma Membrane stain.

Scale bars are 100μm for all images.

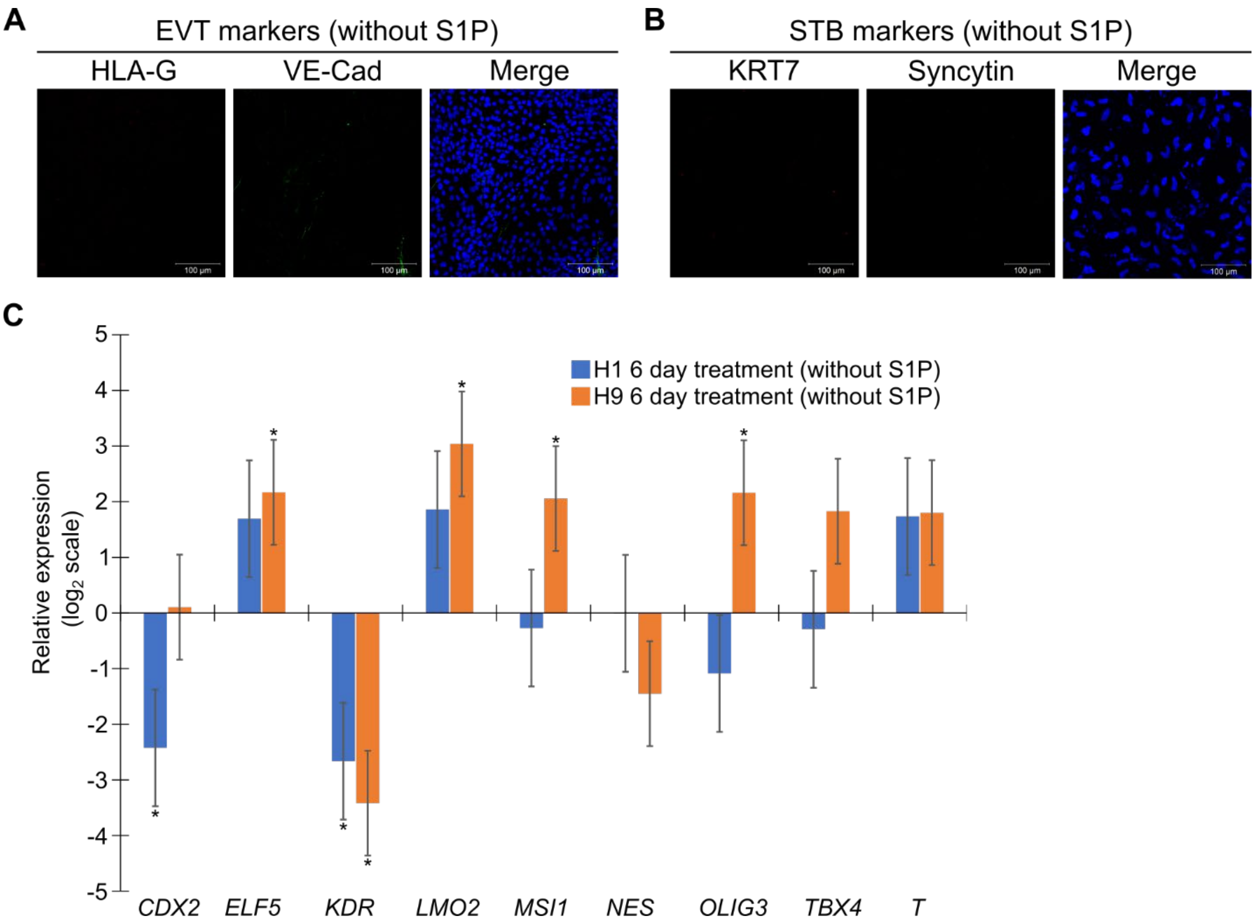

Figure S2: Exogenous S1P is necessary for differentiation of hESCs to trophoblast in chemically defined medium. Related to Figure 1.

(A) Confocal images of cells from 12-day EVT treatment of H1 hESCs upon removal of S1P, staining for HLA-G and VE-Cadherin. Nuclei were stained with DAPI.

(B) Confocal images of cells from 14-day STB treatment of H1 hESCs upon removal of S1P staining for KRT7 and syncytin. Nuclei were stained with DAPI.

(C) Gene expression of *CDX2, ELF5, KDR, LMO2, MSH1, NES* (nestin), *OLIG3*, and *T* (brachyury) in 6-day treatment of H9 and H1 hESCs upon removal of S1P, compared to 6-day treatment in the presence of S1P. Three biological replicates were used. (Error bars are S.E., *$p<0.05$)

Scale bars are 100μm for all images.

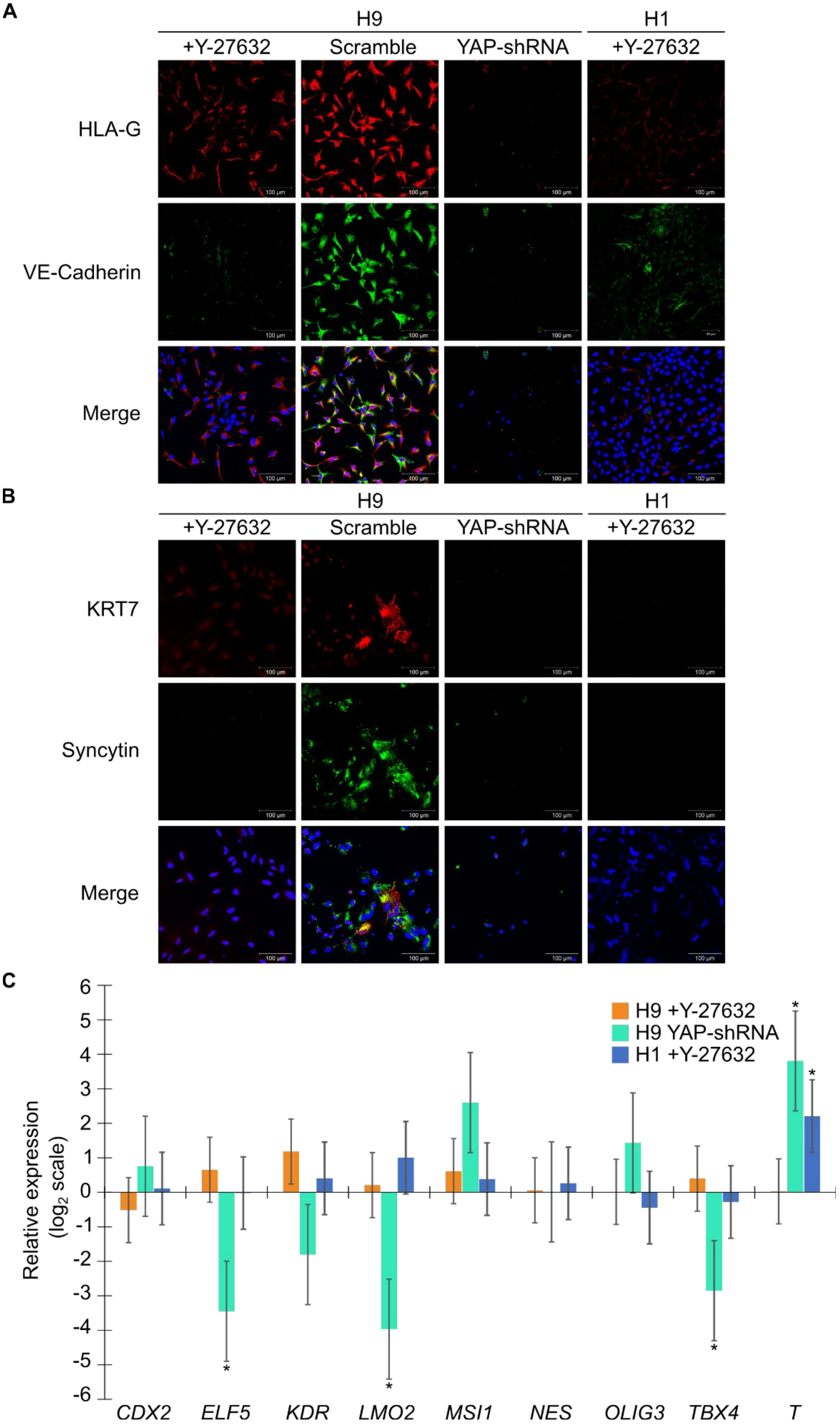
A
H9
H1
+Y-27632
Scramble
YAP-shRNA
+Y-27632
HLA-G
VE-Cadherin
Merge
B
H9
H1
+Y-27632
Scramble
YAP-shRNA
+Y-27632
KRT7
Syncytin
Merge
C
H9 +Y-27632
H9 YAP-shRNA
H1 +Y-27632
Relative expression
(log2 scale)
6
5
4
3
2
1
0
-1
-2
-3
-4
-5
-6
*
CDX2
ELF5
KDR
LMO2
MSI1
NES
OLIG3
TBX4
T

Figure S3: Rho/Rock signaling and YAP are necessary for differentiation of hESCs to trophoblast in chemically defined medium. Related to Figure 1

(A) Confocal images of cells from 12-day EVT treatment of H9 and H1 hESCs with the addition of ROCK inhibitor (+Y-27632), knockdown of YAP using an inducible shRNA (YAP-shRNA), or a scrambled shRNA control (scramble), staining for HLA-G and VE-Cadherin. Nuclei were stained with DAPI.

(B) Confocal images of cells from 14-day STB treatment of H9 and H1 hESCs with addition of ROCK inhibitor (+Y-27632), knockdown of YAP using an inducible shRNA (YAP-shRNA), or a scrambled shRNA control (scramble), staining for KRT7 and syncytin. Nuclei were stained with DAPI.

(C) Gene expression of *CDX2, ELF5, KDR, LMO2, MSH1, NES* (nestin), *OLIG3*, and *T* (brachyury) in H9 and H1 hESCs undergoing differentiation with the addition of ROCK inhibitor (+Y-27632) and knockdown of YAP using an inducible shRNA (YAP-shRNA), compared to the 6-day time point in the presence of S1P or scrambled shRNA knockdown in presence of S1P. Three biological replicates were used. (Error bars are S.E., $^{*}p<0.05$)

Scale bar is 100μm for all images.

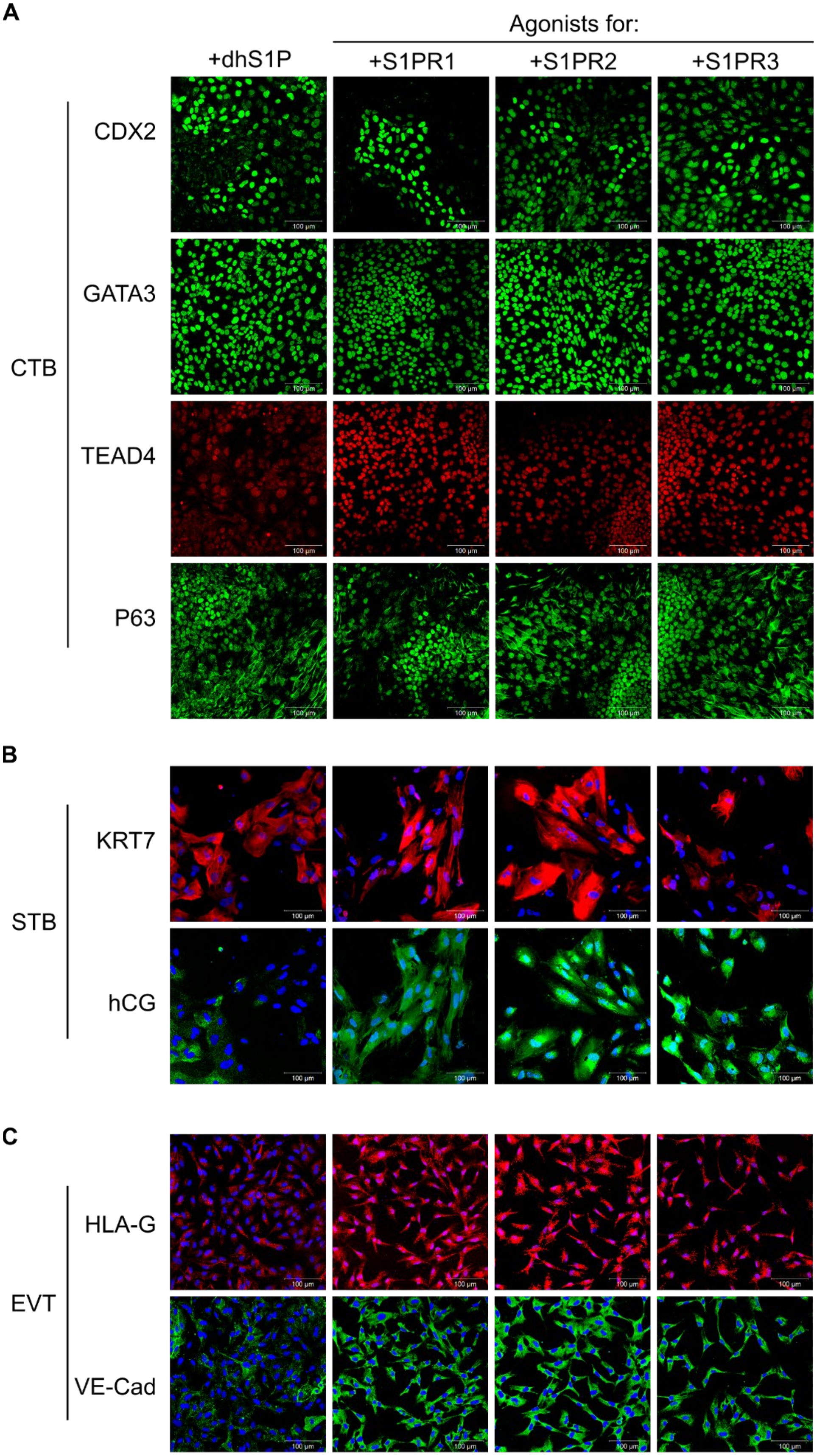
A
Agonists for:
+dhS1P
+S1PR1
+S1PR2
+S1PR3
CTB
CDX2
GATA3
TEAD4
P63
B
STB
KRT7
hCG
C
EVT
HLA-G
VE-Cad
100 µm

Figure S4: S1P mediates its effects on trophoblast differentiation of hESCs through its receptors. Related to Figure 2.

(A) Confocal images of CTB from 6-day treatment of H1 hESCs using D-erythro-dihydrospingosine-1-phosphate (dhS1P), CYM5442 (S1PR1 agonist), CYM5220 (S1PR2 agonist), and CYM5541 (S1PR3 agonist), staining for CDX2, GATA3, P63, and TEAD4. Nuclei were stained with DAPI.

(B) Confocal images of STB from 14-day treatment of H1 hESCs using dhS1P, CYM5442, CYM5520, and CYM5541 during initial 6-day treatment, staining for KRT7 and hCG. Nuclei were stained with DAPI.

(C) Confocal images of EVTs from 12-day treatment of H1 hESCs using dhS1P, CYM5442, CYM5220, and CYM5541 during initial 6-day treatment, staining for HLA-G and VE-Cadherin. Nuclei were stained with DAPI.

Scale bars are 100μm for all images.

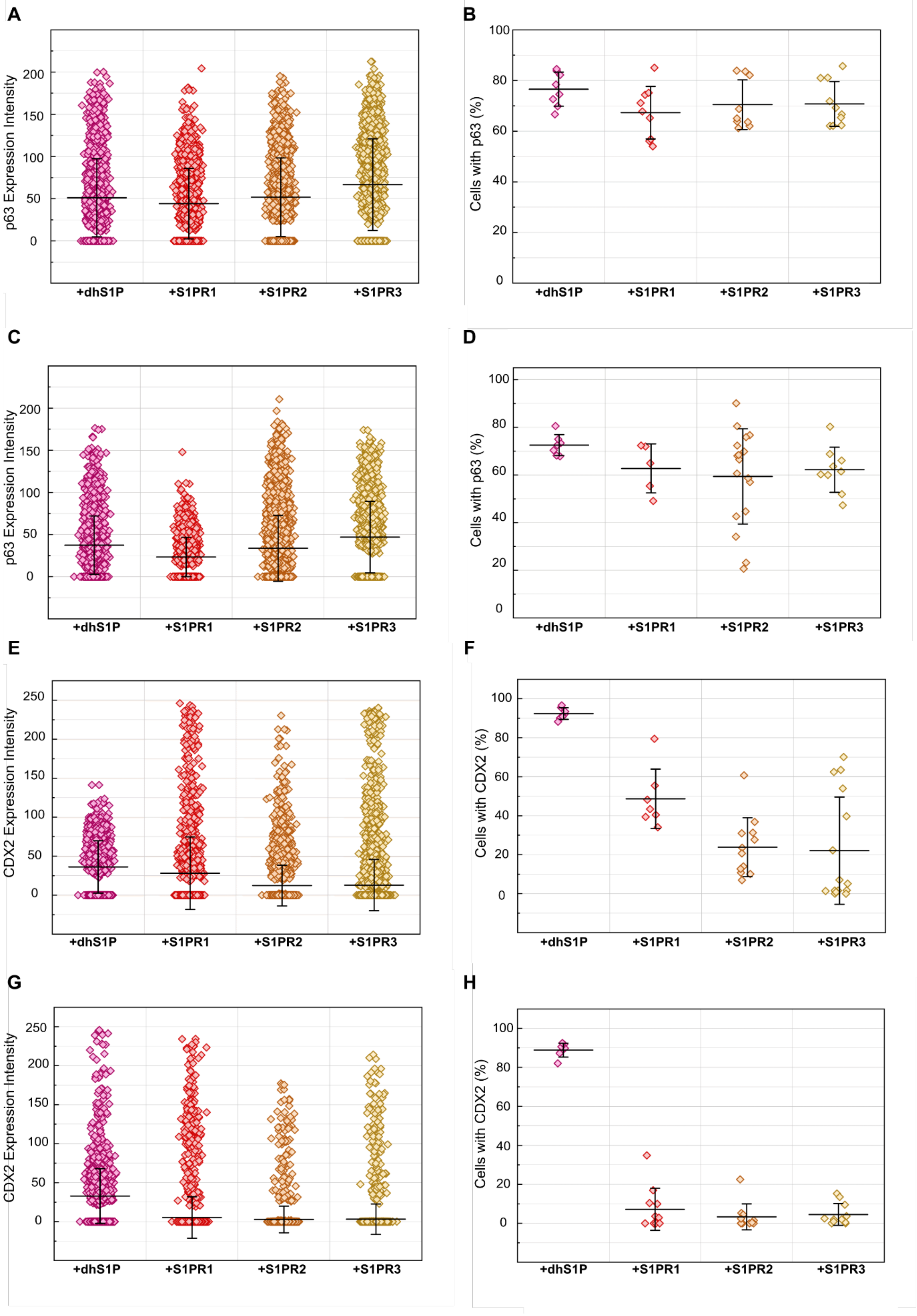
A
p63 Expression Intensity
+dhS1P
+S1PR1
+S1PR2
+S1PR3
B
Cells with p63 (%)
C
p63 Expression Intensity
D
Cells with p63 (%)
E
CDX2 Expression Intensity
F
Cells with CDX2 (%)
G
CDX2 Expression Intensity
H
Cells with CDX2 (%)

Figure S5: S1P mediates its effects on trophoblast differentiation of hESCs through its receptors. Related to Figure 2.

(A) Quantitative analysis of relative intensity of p63 staining after 6-day treatment of H9 hESCs using dhS1P (n=1655 cells in 8 images), CYM5542 (n=2229 cells in 9 images), CYM 5520 (n=2360 cells in 9 images), and CYM 5541 (n=2778 cells in 10 images). Data points represent fluorescence intensity in individual cells. Analysis was performed in MATLAB and at least 2 biological replicates were used. (Error bars are S.D.)

(B) Quantitative analysis of cells expressing p63 after 6-day treatment of H9 hESCs using dhS1P (n=1655 cells in 8 images), CYM5542 (n=2229 cells in 9 images), CYM 5520 (n=2360 cells in 9 images), and CYM 5541 (n=2778 cells in 10 images). Data points represent fraction of $p63^{+}$cells in individual images. Analysis was performed in MATLAB and at least 2 biological replicates were used. (Error bars are S.D.)

(C) Quantitative analysis of relative intensity of p63 staining after 6-day treatment of H1 hESCs using dhS1P (n=2232 cells in 7 images), CYM5542 (n=1471 cells in 5 images), CYM 5520 (n=5768 cells in 17 images), and CYM 5541 (n=26244 in 9 images). Data points represent fluorescence intensity in individual cells. Analysis was performed in MATLAB and at least 2 biological replicates were used. (Error bars are S.D.)

(D) Quantitative analysis of cells expressing p63 of 6-day treatment of H1 hESCs using dhS1P (n=2232 cells in 7 images), CYM5542 (n=1471 cells in 5 images), CYM 5520 (n=5768 cells in 17 images), and CYM 5541 (n=26244 in 9 images). Data points represent fraction of $p63^{+}$cells in individual images. Analysis was performed in MATLAB and at least 2 biological replicates were used. (Error bars are S.D.)

(E) Quantitative analysis of relative intensity of CDX2 staining after 6-day treatment of H9 hESCs using dhS1P (n=2639 cells in 7 images), CYM5542 (n=1872 cells in 7 images), CYM 5520 (n=4539 cells in 12 images), and CYM 5541 (n=5927 cells in 15 images). Data points represent fluorescence intensity in individual cells. Analysis was performed in MATLAB and at least 2 biological replicates were used. (Error bars are S.D.)

(F) Quantitative analysis of cells expressing CDX2 after 6-day treatment of H9 hESCs using dhS1P (n=2639 cells in 7 images), CYM5542 (n=1872 cells in 7 images), CYM 5520 (n=4539 cells in 12 images), and CYM 5541 (n=5927 cells in 15 images). Data points represent fraction of $CDX2^{+}$cells in individual images. Analysis was performed in MATLAB and at least 2 biological replicates were used. (Error bars are S.D.)

(G) Quantitative analysis of relative intensity of CDX2 staining after 6-day treatment of H1 hESCs using dhS1P (n=3425 cells in 7 images), CYM5542 (n=4373 cells in 11 images), CYM 5520 (n=3367 cells in 11 images), and CYM 5541 (n=3839 cells in 11 images). Data points represent fluorescence intensity in individual cells. Analysis was performed in MATLAB and at least 2 biological replicates were used. (Error bars are S.D.)

(H) Quantitative analysis of cells expressing CDX2 of 6-day treatment of H1 hESCs using dhS1P (n=3425 cells in 7 images), CYM5542 (n=4373 cells in 11 images), CYM 5520 (n=3367 cells in 11 images), and CYM 5541 (n=3839 cells in 11 images). Data points represent fraction of

$CDX2^+$cells in individual images. Analysis was performed in MATLAB and at least 2 biological replicates were used. (Error bars are S.D., n represents an individual image).

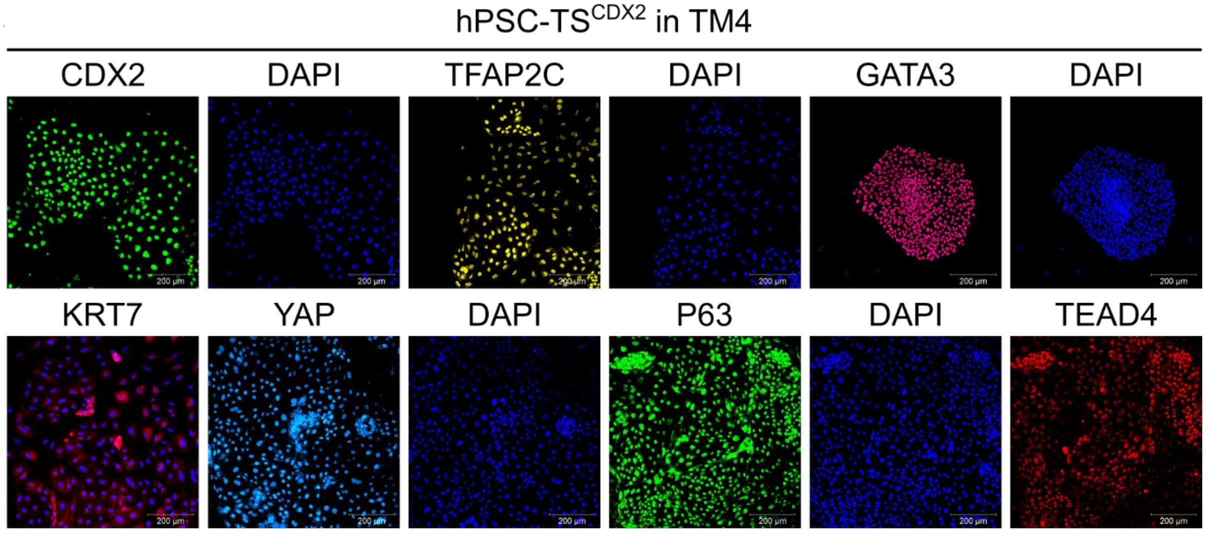

Figure S6: Optimizing timing of hESC differentiation enables derivation of hPSC-TS^{CDX2} cells. Related to Figure 3.

(A) Confocal images of H1 hPSC-TS^{CDX2} in TM4, staining for CDX2, TFAP2C and GATA3, YAP, TEAD4, and P63. Nuclei were stained with DAPI. Scale bars are 200 μm.

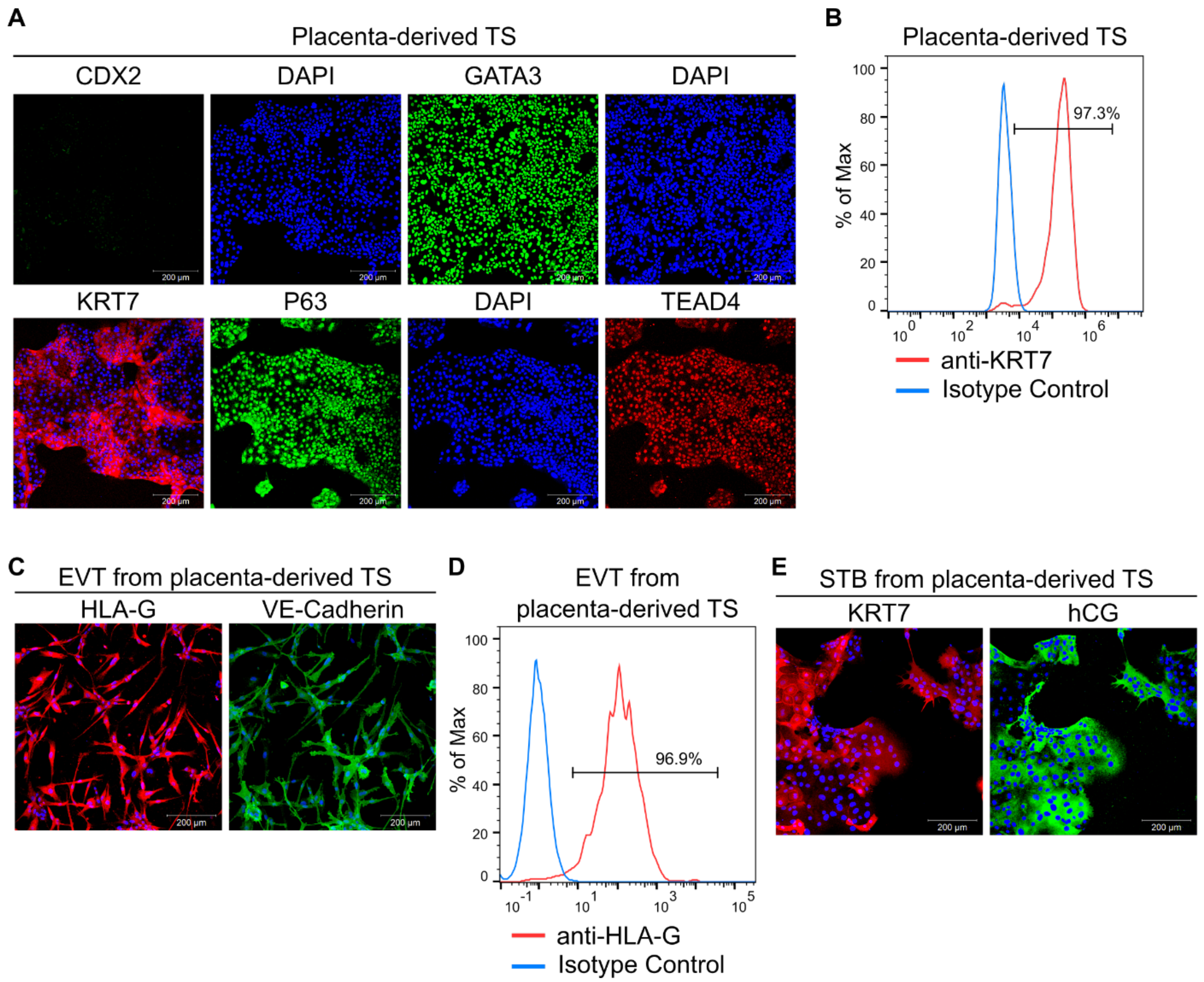

Figure S7: Placenta-derived TS cells. Related to Figure 4.

(A) Confocal images of primary-derived TS cells in TSCM, staining for CDX2, GATA3, TEAD4, and P63. Nuclei were stained with DAPI. Similar results were obtained with another placenta-derived TS cell line. Scale bars.

(B) Flow cytometry histogram of KRT7 expression of primary-derived TS cells compared to isotype control. Similar results were obtained with another placenta-derived TS cell line.

(C) Confocal images of EVTs from primary-derived TS cells, staining for HLA-G and VE-Cadherin. Nuclei were stained with DAPI. Similar results were obtained with another placenta-derived TS cell line. Scale bars are 200μm.

(D) Flow cytometry histogram of HLA-G expression of EVTs from primary-derived TS cells compared to isotype control. Similar results were obtained with another placenta-derived TS cell line.

(E) Confocal images of STB from primary-derived TS cells, staining for KRT7 and hCG. Nuclei were stained with DAPI. Similar results were obtained with another placenta-derived TS cell line. Scale bars are 200μm.

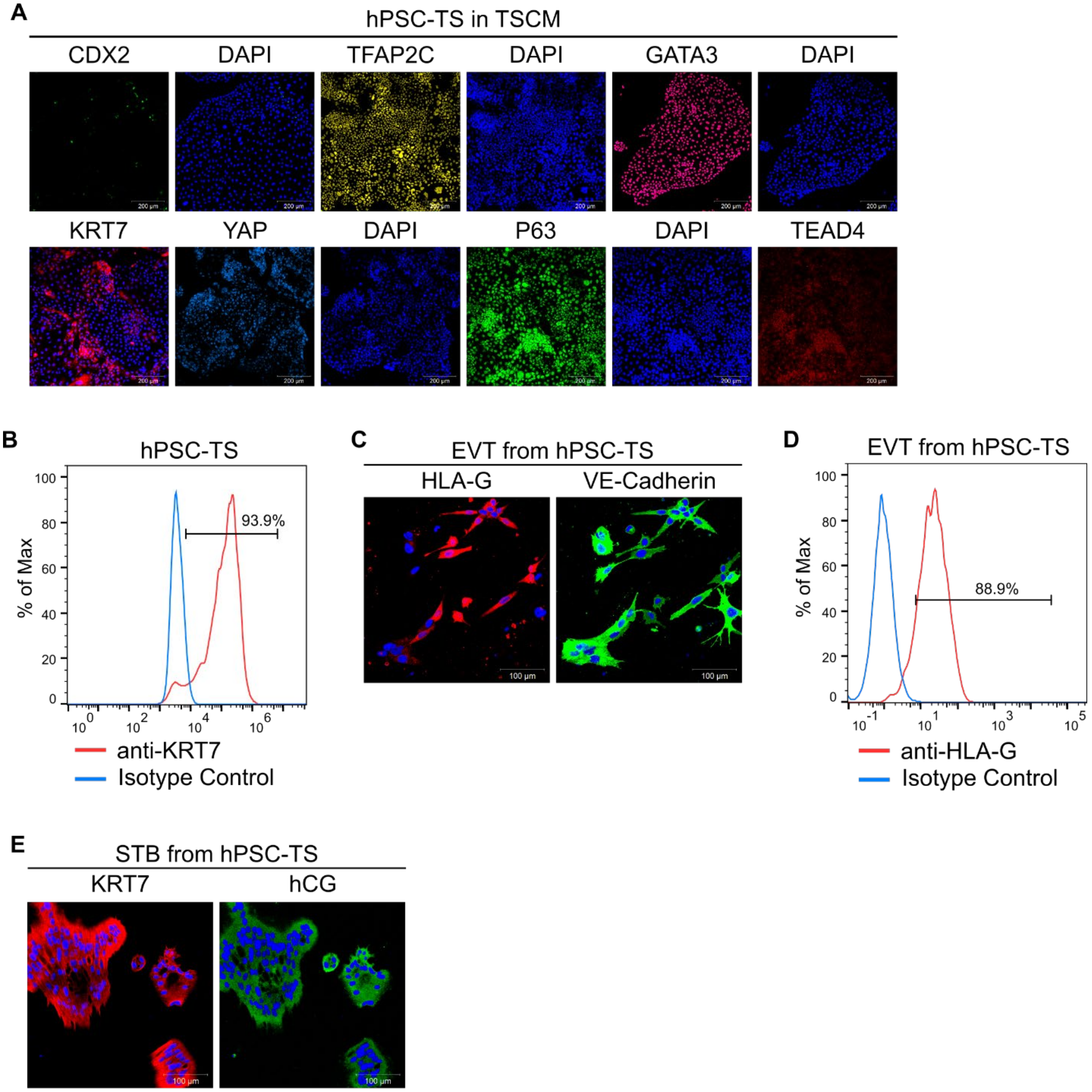

Figure S8: Formation of hPSC-TS cells. Related to Figure 4.

(A) Confocal images of H1 hPSC-TS in TSCM, staining for CDX2, TFAP2C and GATA3, YAP, TEAD4, and P63. Nuclei were stained with DAPI. Scale bars are 200 μm.

(B) Flow cytometry histogram of KRT7 expression of H1 hPSC-TS cells in TSCM compared to isotype control.

(C) Confocal images of EVTs from H1 hPSC-TS cells, staining for HLA-G and VE-Cadherin. Nuclei were stained with DAPI. Scale bars are 100 μm.

(D) Flow cytometry histogram of HLA-G expression of EVTs from H1 hPSC-TS cells compared to isotype control.

(E) Confocal images of STB from H1 hPSC-TS cells, staining for hCG and KRT7. Nuclei were stained with DAPI. Scale bars are 100μm.

Methods S1: List of primers used for quantitative PCR analysis

Gene	Primer	Sequence
CDX2	Forward	GGC AGC CAA GTG AAA ACC AG
CDX2	Reverse	GGT GAT GTA GCG ACT GTA GTG AA
CGB	Forward	CAG CAT CCT ATC ACC TCC TGG T
CGB	Reverse	CTG GAA CAT CTC CAT CCT TGG T
CSH1/2	Forward	CAT GAC TCC CAG ACC TCC TTC T
CSH1/2	Reverse	ATT TCT GTT GCG TTT CCT CCA T
ELF5	Forward	GCT GCG ACC AGT ACA AGT TG
ELF5	Reverse	CTG CCT CGA CGA ACT CCT C
GAPDH	Forward	CTC CAC GAC GTA CTC AGC G
GAPDH	Reverse	TGT TGC CAT CAA TGA CCC CTT
HLA-G	Forward	CCA CCA CCC TGT CTT TGA CTA T
HLA-G	Reverse	ACG TCC TGG GTC TGG TCC T
KDR	Forward	GGC CCA ATA ATC AGA GTG GCA
KDR	Reverse	CCA GTG TCA TTT CCG ATC ACT TT
LMO2	Forward	GGC CAT CGA AAG GAA GAG CC
LMO2	Reverse	GGC CCA GTT TGT AGT AGA GGC
MMP2	Forward	TGG CAC CCA TTT ACA CCT ACA C
MMP2	Reverse	ATG TCA GGA GAG GCC CCA TAG A
MSI1	Forward	TAA AGT GCT GGC GCA ATC G
MSI1	Reverse	TCT TCT TCG TTC GAG TCA CCA
NES	Forward	CTG CTA CCC TTG AGA CAC CTG
NES	Reverse	GGG CTC TGA TCT CTG CAT CTA C
OLIG3	Forward	AGC CGT CTC AAC TCG GTC T
OLIG3	Reverse	CAT GGC TAG GTT CAG GTC GTG
SDC1	Forward	CTA TTC CCA CGT CTC CAG AAC C
SDC1	Reverse	GGA CTA CAG CCT CTC CCT CCT T
T	Forward	CTG GGT ACT CCC AAT GGG G
T	Reverse	GGT TGG AGA ATT GTT CCG ATG A
TBX4	Forward	TGT TCC CCA GCT ACA AGG TAA
TBX4	Reverse	GCA GGG ACA ATG TCA ATC AGC
TEAD4	Forward	CAG GTG GTG GAG AAA GTT GAG A
TEAD4	Reverse	GTG CTT GAG CTT GTG GAT GAA G
TP63	Forward	AGA AAC GAA GAT CCC CAG ATG A
TP63	Reverse	CTG TTG CTG TTG CCT GTA CGT T
